# Metal‐based smart nanosystems in cancer immunotherapy

**DOI:** 10.1002/EXP.20230134

**Published:** 2024-03-22

**Authors:** Ying Luo, Xiaojing He, Qianying Du, Lian Xu, Jie Xu, Junrui Wang, Wenli Zhang, Yixin Zhong, Dajing Guo, Yun Liu, Xiaoyuan Chen

**Affiliations:** ^1^ Department of Radiology Second Affiliated Hospital of Chongqing Medical University Chongqing People's Republic of China; ^2^ Department of Diagnostic Radiology Yong Loo Lin School of Medicine National University of Singapore Singapore Singapore; ^3^ Clinical Imaging Research Centre Centre for Translational Medicine Yong Loo Lin School of Medicine National University of Singapore Singapore Singapore; ^4^ Nanomedicine Translational Research Program NUS Center for Nanomedicine Yong Loo Lin School of Medicine National University of Singapore Singapore Singapore; ^5^ Department of Surgery Chemical and Biomolecular Engineering and Biomedical Engineering Yong Loo Lin School of Medicine and College of Design and Engineering National University of Singapore Singapore Singapore; ^6^ Institute of Molecular and Cell Biology Agency for Science, Technology, and Research (A*STAR) Singapore Singapore

**Keywords:** cancer immunity cycle, immunotherapy, metal ions, smart nanosystem, tumor microenvironment

## Abstract

Metals are an emerging topic in cancer immunotherapy that have shown great potential in modulating cancer immunity cycle and promoting antitumor immunity by activating the intrinsic immunostimulatory mechanisms which have been identified in recent years. The main challenge of metal‐assisted immunotherapy lies in the fact that the free metals as ion forms are easily cleared during circulation, and even cause systemic metal toxicity due to the off‐target effects. With the rapid development of nanomedicine, metal‐based smart nanosystems (MSNs) with unique controllable structure become one of the most promising delivery carriers to solve the issue, owing to their various endogenous/external stimuli‐responsiveness to release free metal ions for metalloimmunotherapy. In this review, the state‐of‐the‐art research progress in metal‐related immunotherapy is comprehensively summarized. First, the mainstream mechanisms of MSNs‐assisted immunotherapy will be delineated. The immunological effects of certain metals and categorization of MSNs with different characters and compositions are then provided, followed by the representative exemplar applications of MSNs in cancer treatment, and synergistic combination immunotherapy. Finally, we conclude this review with a summary of the remaining challenges associated with MSNs and provide the authors' perspective on their further advances.

## Graphical abstract

Metal‐based smart nanosystems show great potential in modulating each phase of the cancer immunity cycle to boost anticancer immune responses against tumor growth, recurrence, and metastasis mainly by inducing immunogenic cell death, repolarizing protumoral M2 tumor associated macrophages(TAMs) into antitumoral M1 phenotype, and activating the cGAS‐STING signaling pathway.

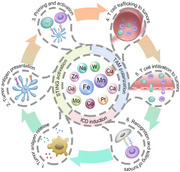

## 1 INTRODUCTION

Immunotherapy, an evolving therapeutic paradigm for cancer, has gained intense attention during the last few decades and holds great promise to overcome the lack of specificity related to the current chemotherapy, radiotherapy, and surgery approaches.^[^ ^1^ ^]^ Immunotherapy exploits the body's innate and adaptive immune systems to ward off cancer cells.^[^ ^2^ ^]^ Specifically, immune effector cells can travel through the body, identify metastatic tumor cells and destroy them. Moreover, immune memory cells are capable of killing recurrent tumor cells, thereby preventing tumor relapse.

Mainstream immunotherapy approaches can be categorized into immune checkpoint blockades (ICBs), adoptive cell therapies (ACTs), cancer vaccines, and non‐specific immune adjuvants.^[^ ^3^ ^]^ However, the overall clinical response rates are greatly limited due to the complexity of the immune tumor microenvironment (TME) and the immune escape mechanism developed by tumor cells.^[^ ^4^ ^]^ On the one hand, most current immunotherapies target just one or two types of immune cells and neglect the crosslinked immune networks that comprise various immune cells and soluble factors which interact with each other in multiple ways. On the other hand, tumor cells can escape from immune cell surveillance through various means, thus compromising the efficacy of immunotherapies.^[^ ^5^ ^]^ As an example, ICBs, such as anti‐PD1/L1 antibodies or anti‐CTLA‐4 antibodies, rescue exhausted T cells and unleash their cytotoxic abilities by blocking co‐inhibitory signals.^[^ ^6^ ^]^ Nonetheless, only moderately exhausted T cells can be saved, while highly exhausted T cells cannot.^[^ ^7^ ^]^ Hence, Looking for new approaches that can alleviate tumor immunosuppression and block tumor immune escape mechanism can help enhance the treatment outcome of current immunotherapies.

**FIGURE 1:**
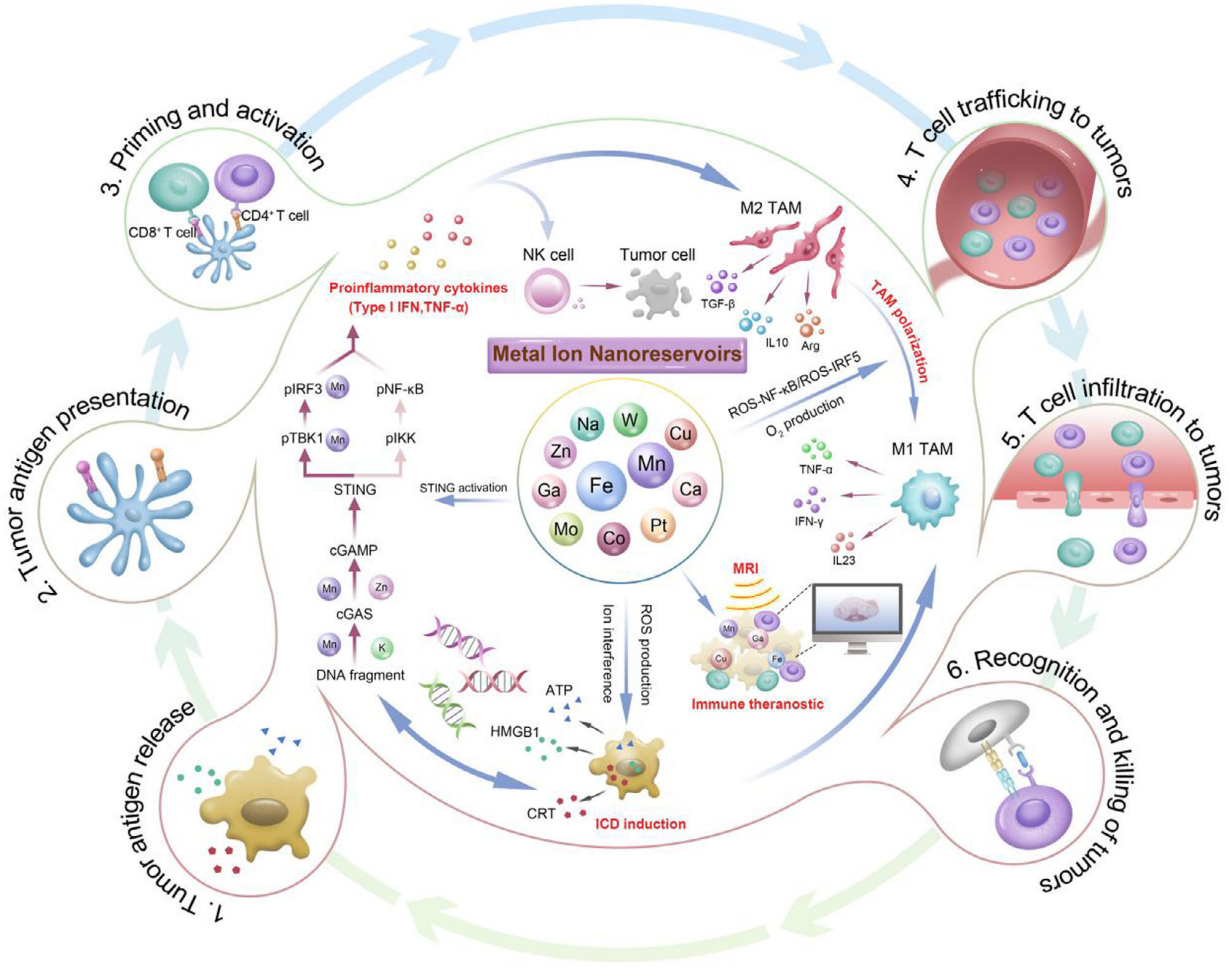
The schematic illustration of the three well‐defined antitumor immunogenic pathways mediated by metals, including STING activation, immunogenic cell death induction, and tumor associated macrophages repolarization. More specifically, the imaging characteristics of some metal elements, including Gd, Fe, Mn, and Cu, enables immune theranostic purposes.

In recent years, the immunostimulatory effects of metal ions have been enthusiastically explored for enhancing the efficacy of immunotherapy.^[^ ^8^ ^]^ Metal elements are present in the body as functional moieties, nutrients, and trace elements at a low level, which would not pose a health threat to the body.^[^ ^9^ ^]^ Excitingly, scientists found that some metal elements participate in some major steps of cancer immunity cycle (CIC), including antigen processing and presentation, T cell priming and activation, infiltration of T cells into tumors, immune recognition of cancer cells, killing of cancer cells, and antigen release. The schematic illustration of the relationship between the CIC and metal element‐induced antitumor immunogenicity was shown in Figure 1. Mechanistically, the specific valences of Fe, Mn, Cu, Co, Zn, Ca, Pt, W etc. can induce Fenton, Fenton‐like reactions or other biological signaling pathways to produce excessive reactive oxygen species (ROS) and induce endothelial reticulum (ER) stress, thereby inducing immunogenic cell death (ICD) for activating the innate and adaptive immune systems to destroy cancer cells.^[^ ^10^ ^]^ In addition to ROS production, Fe, Mn, and Zn can stimulate some specific signaling pathways to exert antitumor immunity.^[^ ^11^ ^]^ Fe can activate IRF5 or NF‐κB signaling pathways for macrophage polarization.^[^ ^11d^ ^]^ Furthermore, Mn^2+^ and Zn^2+^ can boost the activation of cyclic GMP‐AMP synthase/stimulator of interferon genes (cGAS/STING) signaling pathway, leading to the production of type I interferons (IFNs) which induce dendritic cell (DC) maturation, antigen presentation, natural killer (NK) cell activation, and even macrophage polarization.^[^ ^11a,b,e^ ^]^ Ca^2+^ can not only promote the release of damage‐associated molecular patterns (DAMPs) from dying tumor cells by disrupting mitochondrial function but also disrupt autophagy inhibition condition in DCs, thereby improving DC maturation and antigen presentation to naïve T cells.^[^ ^12^ ^]^ Excess amount of intracellular Na^+^ can cause a surge of osmolarity and lead to caspase‐1‐mediated pyroptosis which is also a manner of ICD, leading to immune activation.^[^ ^13^ ^]^ This list goes on and on.

Hence, the great antitumor potential of metals may be hopeful to assist in current mainstream immunotherapies. However, free metals face hindrances in clinical use, such as rapid clearance, off‐target effect, and non‐specific absorption in major organs, leading to systemic metal toxicity.^[^ ^14^ ^]^ Metal‐based drugs, such as Pt‐incorporated drugs (cisplatin and oxaliplatin) and Cu‐incorporated drugs (Elesclomol), are used to treat various types of cancer and play significant roles in combination therapies.^[^ ^15^ ^]^ Specifically, some of these metallodrugs can induce ICD and stimulate various immune‐related pathways to inhibit tumor growth.^[^ ^16^ ^]^ However, metal‐based drugs also face various limitations in clinical settings, including poor bioavailability, low target selectivity, short circulation, metal‐related off‐target toxicity, and development of tumor drug resistance.

Nanomedicines have drawn increasing interest among scientific communities since nanomaterials can deliver therapeutic agents into the targeted tumor sites.^[^ ^17^ ^]^ Furthermore, some special nanomaterials possess intrinsic stimuli responsiveness, such as photothermal, magnetothermal, photodynamic, sonodynamic and X‐ray responsive properties, thereby initiating various physical or chemical therapies concurrently.^[^ ^18^ ^]^ Therefore, metal‐based smart nanosystems (MSN) may address the aforementioned shortcomings of free metals and metallodrugs and open a new avenue for cancer immunotherapy. In response to specific chemical stimuli (pH and redox signals) in the TME, metal ions can be released from these nanomaterials responsively, thereby realizing theranostic purposes.

Admittedly, metalloimmunotherapy may not overcome all the dilemmas associated with tumor immunosuppression and therapy resistance, it is still of significance to make a comprehensive summary and systematic classification of current research that are related to metalloimmunotherapy, thereby providing new thoughts for oncologists and potential alternatives for current mainstream immunotherapies with suboptimal clinical response rates. The purpose of this review is to highlight the breakthroughs in cancer metalloimmunotherapy. In this review, we systematically introduced the immunological effects of certain metals, categorized their nano‐related materials, and lastly pointed out the potential of metal element‐based smart nanosystems in immunotheranostic applications.

## 2 IMMUNOLOGICAL EFFECTS OF CERTAIN METALS AND THEIR NANO‐RELATED MATERIALS

Certain metal elements with elevated content in tumor microenvironment, such as Mn, Fe, Cu, Ca, Zn, Na, Gd, Pt, K, W, and Co, can activate innate and adaptive immune systems to inhibit tumor growth through multiple mechanisms. Even though metal element‐assisted cancer immunotherapies have been enthusiastically explored in recent years, the mechanisms of which remain largely unknown. Furthermore, introducing overdosed free metals into living bodies would pose unexpected health threats. Thus, numerous nano‐related materials were employed to serve as smart metal nanosystems to deliver free metal ions and release them in the TME in a stimuli‐responsive manner. Herein, we summarized some well‐known immune‐related metal elements, key immune‐related pathways involved within each metal element, and nano‐related materials that can serve as metal nanoreservoirs.

### 2.1 Manganese (Mn)

Mn, which is one of the most abundant trace elements within mammals, was frequently found to be involved in multiple regulatory pathways in both innate and adaptive immunity via inducing ICD, activating cGAS‐STING pathway, and repolarizing TAMs. In this section, we summarized the major signaling pathways participated by Mn in antitumor immune responses and introduced Mn‐involved nanomaterials that can assist in delivering Mn element into the tumor site precisely without inducing systemic metal toxicity.

#### 2.1.1 Immune‐related pathways

##### STING pathways

**FIGURE 2:**
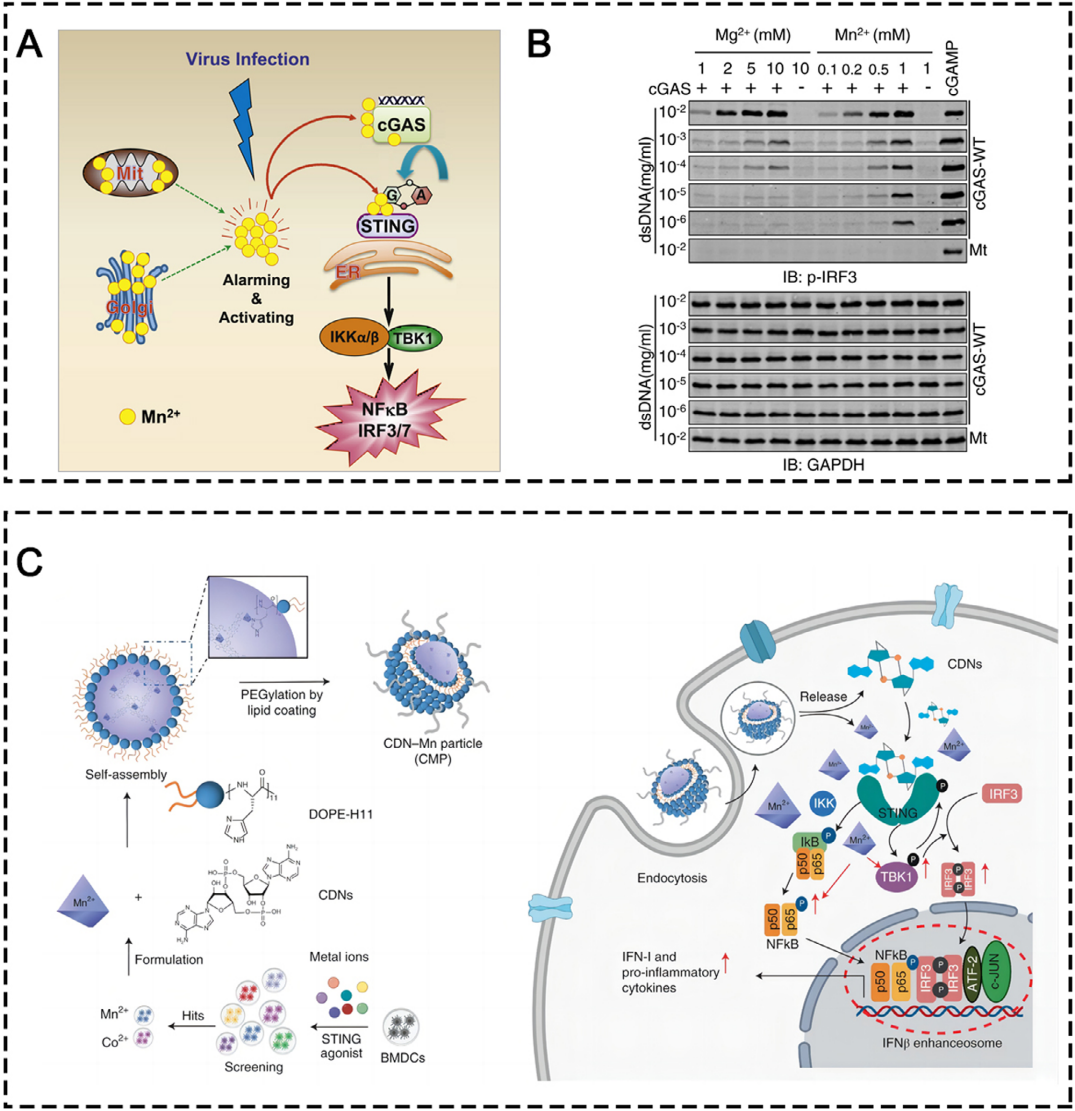
(A) Schematic illustration of Mn^2+^‐activated cGAS‐STING signaling pathway against DNA virus. (B) Western blot analysis of cGAMP‐induced IRF3 phosphorylation. cGAMP was produced by cGAS (wild‐type, WT; or E225A/D227A mutant, Mt) in the presence of the indicated amounts of dsDNA, MnCl_2_, or MgCl_2_. Reprinted with permission.^[^ ^11e^ ^]^ Copyright 2018, Elsevier Inc. (C) Schematic illustration of the mechanism of CMPs‐induced cGAS‐STING activation. Reprinted with permission.^[^ ^21^ ^]^ Copyright 2021, Springer Nature.

In 2018, Mn was first reported as a potent stimulator of the cGAS/STING signaling pathway to stimulate the innate and adaptive immune system to fight against DNA viruses by enhancing the ligand sensitivity of cGAS for dsDNA, increasing its enzymatic activity for production of secondary messenger cGAMP at low concentration of dsDNA, and augmenting cGAMP‐STING binding affinity (Figure 2A,B).^[^ ^11e^ ^]^ Specifically, they found that Mn^2+^ itself could induce type I IFN responses and cytokine production without any infection. Moreover, Mn^2+^‐induced ROS production and mitochondrial damage were shown to be not related to the process of cGAS‐STING activation. The mechanism of Mn^2+^‐induced cGAS‐STING signaling activation was addressed by Hooy and co‐workers.^[^ ^19^ ^]^ They answered the question of how Mn^2+^ potentiated the dsDNA sensing activity of cGAS. In their study, they proved that Mn^2+^ not only increased the catalytic activity of monomeric cGAS directly but also allosterically enhanced its dsDNA binding activity in conjunction with ATP/GTP substrates with no dependence on dsDNA length. Furthermore, they found that Mn^2+^ coupled with dsDNA‐binding maximized the activation of STING signaling and the production of Type I IFN in a concerted manner.

Even though the above studies only explored the defensive role of Mn^2+^ against DNA viruses, the implication of Mn^2+^‐activated cGAS‐STING signaling pathway in cancer immunotherapy is prominent.^[^ ^20^ ^]^ Later on, Mn^2+^ was further demonstrated to initiate strong antitumor immune responses by promoting antigen presenting cell (APC) maturation, increasing CD8^+^ T cell, NK cell, and memory T cell frequencies in a cGAS‐STING‐dependent manner.^[^ ^11a^ ^]^ In 2021, Sun and coworkers coordinated Mn^2+^ with STING agonists to develop CDN‐Mn^2+^ NPs (CMPs). Robust antitumor immunity elicited by CMPs was evidenced in multiple murine tumor models.^[^ ^21^ ^]^ In this study, they found something contradictory to the report from a previous study that proposed that Mn^2+^ augmented cGAMP‐STING binding affinity. They found that Mn^2+^ did not increase the binding affinity between STING and STING agonists. Instead, Mn^2+^ in tandem with STING agonists phosphorylated TBK1, IRF3, and p65 in the downstream of the STING pathway, leading to amplified type I IFN production (Figure 2C). To sum up, the mechanism of antitumor immunogenicity induced by Mn^2+^‐mediated STING activation can be classified into the following unique ways: (i) Mn^2+^ independently activates monomeric cGAS in the absence of dsDNA; (ii) Mn^2+^ enhances the dsDNA‐binding capacity of cGAS, lowering the threshold for STING pathway activation; (iii) Mn^2+^ accelerates the overall catalytic activity of dsDNA‐bound cGAS, resulting in much greater production of cGAMP; (iv) Mn^2+^ induces the phosphorylation of both TBK1 and p65 in a STING‐independent manner, and when in the presence of STING agonists, leading to the formation of an enhanceosome which contains phosphorylated IRF3 and p65, resulting in greatly increased production of type I IFN.^[22]^

##### ICD pathways

It is well known that metal element in a low valence state that possess peroxidase (POD)‐like activities, help in the decomposition of H_2_O_2_ into hydroxyl radical (•OH) under an acidic environment, while metal ions in a high valence state that possess catalase (CAT)/glutathione peroxidase (GPx)‐like activities, promote O_2_ production and glutathione (GSH) degradation into oxidized glutathione disulfide (GSSG), which assist in alleviating intratumoral hypoxia and preventing ROS elimination.

In biological systems, Mn that cycles between Mn^2+^ and Mn^4+^ can catalyze the production of ROS via Fenton‐like reaction and preventing ROS elimination due to GPx‐like activities. Excessive cellular ROS leads to tumor ICD by inflicting damage on DNAs in the nuclei and proteins on subcellular organelles, thereby stimulating the release of tumor TAAs, neoantigens, DAMPs, and pro‐inflammatory cytokines, which can possibly act as adjuvants for recruiting and activating APCs, thus causing antigen presentation and immune effector cell infiltration in various tumors.

##### TAM repolarization

Materials that can produce ROS, including H_2_O_2,_ superoxide radical (O^2−^), •OH, and OONO^−1^, have been demonstrated to effectively activate a classical inflammatory pathway, that is, TLR/NF‐ĸB signaling pathway.^[^ ^23^ ^]^ The activated NF‐ĸB transcription factor can further promote the gene expression of various inflammatory mediators (TNF‐α, IFN‐γ, IL‐1β, and iNOS), increase the expression of M1‐TAM surface markers (CD80, CD86, and CD163), and down‐regulate the expression of M2‐TAM surface markers (CD206), resulting in an inflammatory TME infiltrated with abundant M1‐like TAMs. Mn with POD‐like activities catalyzes Fenton‐like reactions to produce ROS in tumor cells. Excessive intracellular ROS can not only induce the aforementioned ICD but also repolarize pro‐tumoral M2 TAMs into the tumoricidal M1 phenotype.

Beyond that, Mn with CAT‐like activities has been validated to produce O_2_ in the TME, leading to hypoxia alleviation and thereby TAM repolarization. Various MnO_2_‐based nanocomplexes have been reported to catalyze endogenous H_2_O_2_ into O_2_ for alleviating tumor hypoxia and M1 polarization.^[^ ^24^ ^]^

#### 2.1.2 Mn‐containing nanomaterials

For efficacious tumor destruction, it is critical to deliver large amount of Mn element into the TME, since the intensity of Mn‐triggered immuno‐activities is positively correlated with the Mn content. However, introducing overdosed free Mn element systemically becomes harmful, because Mn in excess can induce uncontrollable oxidative stress by generating free radicals and reducing antioxidant levels in major organs besides tumor sites. Even worse, excessive Mn ions can also alter the confirmation of protein and DNA, thus inhibiting their functions. Therefore, it is necessary to figure out a way to deliver enough Mn element into the tumor site precisely without causing severe systemic toxicity. Nanomedicine seems to be a feasible approach since metal‐related nanomaterials can be tuned to release free metal ions in the TME in response to local stimuli, such as redox signal and acidity. In this section, we categorized the popular Mn‐containing nanomaterials which were applied to elicit antitumor immunity.

##### Inorganic nanomaterials

Metal fixed in inorganic nanomaterials usually maintains a relatively stable state and is not prone to degrade in circulation, thus avoiding premature leakage of free metal ions to non‐target organs. Manganese oxide NPs (MONPs), like MnO, MnO_2_, and MnO_x_, have been constructed for cancer nanomedicine.^[^ ^24^, ^25^ ^]^ MONPs‐induced metalloimmunotherapy mainly lies in four mechanisms: (i) Mn^4+^ reacts with H_2_O_2_ to produce O_2_ for hypoxia alleviation and TAM polarization; (ii) Mn^4+^ reacts with GSH to prevent ROS elimination for amplifying ICD effect; (iii) Mn^2+^ reacts with H_2_O_2_ to produce ROS for CDT and concurrent ICD; (iv) Mn^2+^ directly activates the cGAS‐STING signaling pathway to activate the innate and adaptive immune systems. To maximize the therapeutic effect of MONPs, copolymer materials, like silica, polyethylene glycol (PEG), functional polyphenols, poly acrylic acid (PAA), biomimetic membranes and targeting ligand etc., have been applied to camouflage naked MONPs for better biocompatibility, targeting performance and conjugation of other ingredients for amplifying antitumor effects.

**FIGURE 3:**
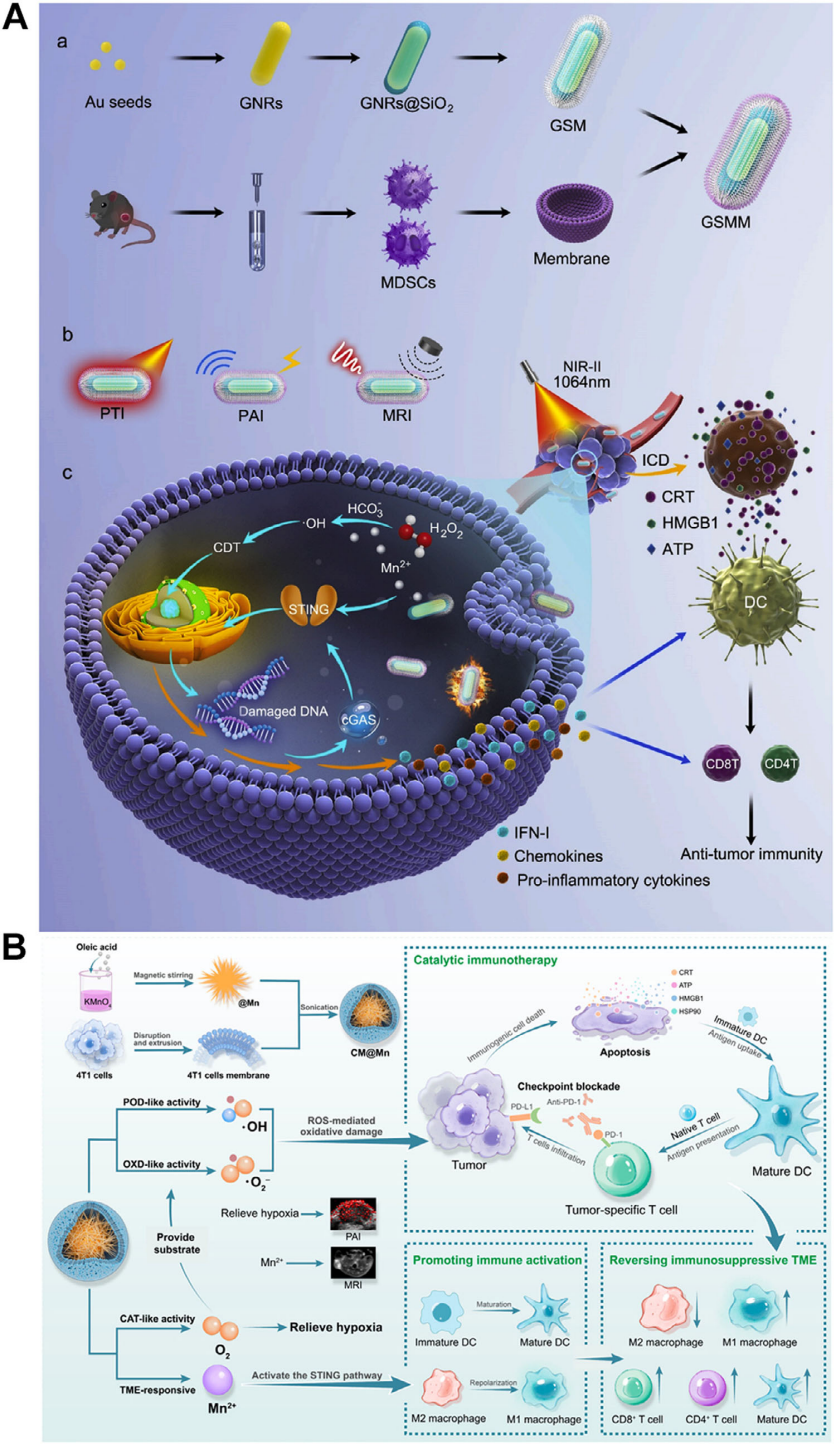
(A) Schematic illustration of the synthetic process of GSMM nanoplatform and the mechanism of GSMM for enhancing anti‐tumor immunity by PTT and STING activation. (GSMM: GNRs@SiO2@MnO2@MDSCs). Reprinted with permission.^[^ ^26^ ^]^ Copyright 2022, Elsevier. (B) The fabrication of CM@Mn NPs and the schematic diagram of Mn‐enhanced catalytic immunotherapy by manipulating MnO_x_ as ICD inducer, hypoxia reducer, and STING activator. Reprinted with permission.^[^ ^28^ ^]^ Copyright 2012, American Chemical Society.

As an example, Zhao et al. explored and exploited the MnO_2_‐mediated crosstalk between ICD and cGAS‐STING activation to destroy melanoma growth (Figure 3A).^[^ ^26^ ^]^ In their study, SiO_2_ was used to coat gold nanorods (GNR), followed by the attachment of MnO_2_ and myeloid‐derived suppressor cells (MDSCs) layer by layer. MDSC membrane allowed homologous targeting for the TME. Mn^2+^ was released into TME in response to intratumoral excessive GSH and H_2_O_2_ and then induced ICD via ROS production. Specifically, GNR‐led photothermal therapy (PTT) synergistically amplified Mn^2+^‐led ICD, and subsequently damaged and exposed cytosol double‐stranded DNA (dsDNA) which was detected by cGAMP synthase (cGAS), thereby activating the cGAS‐STING pathway from the start. Moreover, Mn^2+^ stimulated the downstream of the STING pathway, which further amplified the pathway activation in tandem with ICD‐induced DNA exposure. The interplay between ICD and STING activation was demonstrated to elicit potent melanoma suppression. In another study, the CAT‐like activity of MnO_2_ was utilized to produce O_2_ for TAM polarization.^[^ ^27^ ^]^ Hou *et al.* constructed nanoformulations by integrating doxorubicin (DOX), indocyanine green (ICG), and MnO_2_. In this study, MnO_2_ exerted its immunomodulatory effect by catalyzing O_2_ production to alleviate intratumoral hypoxia, thus polarizing M2‐like TAMs into the M1 phenotype. This TAM‐based antitumor strategy in tandem with ICG‐induced PDT, DOX‐elicited chemotherapy, and anti‐PD‐1 antibody‐induced immunotherapy synergistically induced potent tumor inhibition in Hepa1‐6 tumor mouse models. More recently, cancer cell membrane coated‐MnO_x_ NPs (CM@Mn nanozyme) with a mixed valences of both Mn^2+^ and Mn^3+^ were fabricated by Zhao's group for suppressing 4T1 tumor growth in combination with PD‐1 checkpoint blockade (Figure 3B).^[^ ^28^ ^]^ The hybrid Mn^2+^ and Mn^3+^ valences endowed MnO_x_ with oxidase‐like (OXD‐like), POD‐like, and CAT‐like activities for •O^2−^, ·OH, and O_2_ productions. Furthermore, in response to intratumoral GSH and H_2_O_2_, MnO_x_ was ultimately decomposed into Mn^2+^ which modulated the immunosuppressive TME by cGAS‐STING activation. Therefore, the CM@Mn nanozyme exerted the antitumor therapeutic potential of Mn^2+^ to the full extent by manipulating MnO_x_ as ICD inducer, hypoxia reducer, and STING activator. The CM@Mn nanozyme was proven to significantly elicit ICD as revealed by the elevation of CRT, HMGB1, and HSP90, promote DC maturation by increasing the CD80^+^CD86^+^ DC proportion, induce TAM repolarization by upregulating CD86 and downregulating CD206 markers in TAMs, and recruit CTLs into the tumor center. The CM@Mn nanozyme in combination with PD‐1 checkpoint blockade was further demonstrated to prolong the survival of tumor‐bearing mice, maintain long‐term antitumor immune memory, and inhibit distant 4T1 tumor growth as revealed by survival observation, the elevated frequency of memory T cells and effector CD8^+^ T cells compared to other groups.

Other than MONPs, manganese zinc sulfide NPs, have been employed for cancer metalloimmunotherapy. For example, Li and co‐workers fabricated manganese zinc sulfide nanoparticles (ZMS) for metastatic melanoma immunotherapy (Figure 9A).^[^ ^29^ ^]^ ZMS and IR780 dye were ultrasonically encapsulated into a thermally sensitive amphiphilic polyethylene glycol‐poly(2‐hexoxy‐2‐oxo‐1,3,2‐dioxaphospholane) copolymer (mPEG‐b‐PHEP) for near infrared radiation (NIR)‐responsive drug release. After NIR irradiation, the photothermal conversion of IR780 enables the thermal sensitive release of ZMS for the following Mn^2+^‐induced ROS production, ICD induction, and cGAS‐STING pathway activation, leading to potent antitumor effect with prolonged mouse survival.

Metal carbonates have also been fabricated to fix Mn. Calcium‐doped manganese carbonate microspheres (Ca@MnCO_3_) were fabricated to load perforin‐listeria hemolysin (LLO), denoted as Ca@MnCO_3_/LLO.^[^ ^30^ ^]^ Benefiting from the lysosomal membrane destruction induced by LLO, Ca^2+^ and Mn^2+^ were released into the cytoplasm from the nanocomposite and then initiated strong antitumor immune responses via autophagy promotion and cGAS/STING activation, leading to significant B16‐OVA tumor growth inhibition.

##### Organic–inorganic hybrid nanomaterials

Nanoscale metal phenolic networks (MPNs) are environment friendly since they are composed of green phenolic ligands. Structurally, MPNs are super‐molecular network consisting of metal ions and phenolic ligands, which are endowed with negligible cytotoxicity, pH responsiveness, and even photothermal performance.^[^ ^31^ ^]^ Multivalent metal ions, like Fe and Mn ions, can induce the crosslinking of natural polyphenols (tannic acid (TA)) and artificial polyphenol derivatives (PEG‐polyphenols), leading to the self‐assembly of nanoscale MPNs.

Han and co‐workers constructed MPN NPs that were prepared through coordination bonding between TA and Mn^2+^, followed by CpG‐ODN coating on the NP surface through hydrogen bonding.^[^ ^32^ ^]^ The CpG‐ODN‐coated MPN (CMP) NPs resulted in effective TAM polarization, ICD induction, and STING activation in CT26 colon cancer models. In combination with irreversible electroporation (IRE), the CMP NPs effectively inhibited CT26 tumor growth and significantly improved mouse survival rate. Similarly, Wang's group constructed CDN‐loaded MPNs (TMA‐NPs) by coordinating Mn^2+^ and TA.^[^ ^33^ ^]^ As a STING agonist, c‐di‐AMP, a type of CDN, was combined with Mn^2+^ to activate the cGAS/STING signaling pathway. To maximize the overall therapeutic effect in 4T1 tumor model, X‐ray irradiation was applied to strengthen STING‐mediated cancer immunotherapy by causing a large amount of DNA damage.

Likewise, Li and co‐workers reported that Mn^2+^, STING agonist amidobenzimidazole (ABZI), and photosensitizer 5,9,14,18,23,27,32,36‐octabutoxy‐2,3‐naphthalocyanine (ONc) were self‐assembled into ONc‐Mn‐A which were further decorated with maleimide‐modified Pluronic F127 (malF127) to form stable nano micelles (ONc‐Mn‐A‐malF127).^[^ ^34^ ^]^ The Mn^2+^‐coordinated nano micelles significantly activated the cGAS‐STING signaling pathway and triggered type I IFN response in CT26 tumor‐bearing mice. With a single systemic administration, ONc‐Mn‐A‐malF127 treatment significantly inhibited primary tumor growth with a cure rate of 25%, which was ascribed to immune stimulation by the Mn^2+^‐coordinated nano micelles, including DC maturation, CD4^+^ and CD8^+^ T cell activation, and NK cell stimulation. Remarkably, the addition of laser irradiation completely eradicated both primary and distant CT26 tumors with substantial immune cell infiltration. Sun et al. reported that Mn^2+^ coordinated with c‐di‐AMP self‐assembled into CDA‐Mn^2+^ coordination polymers.^[^ ^33^ ^]^ To stabilize the copolymer, dioleoyl‐sn‐glycero‐3‐phosphoethanolamine‐N [histidine]11 (DOPE‐H11) was introduced to act as an additional coordination ligand and promoted the formation of a hydrophobic core, followed by coating an outer PEG‐lipid layer for aqueous suspension. The nano drug was demonstrated to achieve remarkable therapeutic effect via cGAS‐STING pathway activation in multiple murine tumor models through local or systemic treatment. Similarly, Yang's group coordinated Mn^2+^ with STING agonist, cGAMP, to form a nanovaccine to direct cytosolic co‐delivery of Mn^2+^ and cGAMP, thereby potentiating the activation of cGAS‐STING signaling pathway and leading to suppression of the primary and abscopal tumor growth in poorly immunogenic melanoma mice.^[^ ^35^ ^]^

In another study, Wang et al. loaded Mn^2+^ ions into mannose (Man)‐functionalized BSA (BSA‐Man@Mn^2+^) via a simple ion diffusion method and further crosslinked the BSA‐Man@Mn^2+^ with β‐lapachone‐loaded ferritins via acid‐responsive Schiff base linkers for pH‐responsive dissociation in the acidic TME.^[^ ^36^ ^]^ β‐lapachone was precisely delivered inside the tumor cells via ferritins and subsequently caused ICD and dsDNA outflow into the TME, while Mn ions were delivered into DCs. After DCs sensed exposed dsDNA in the TME, the cGAS‐STING pathway was activated with the help of Mn^2+^, ultimately leading to intensive antitumor immune responses in a poorly immunogenic 4T1 tumor model.

**FIGURE 4:**
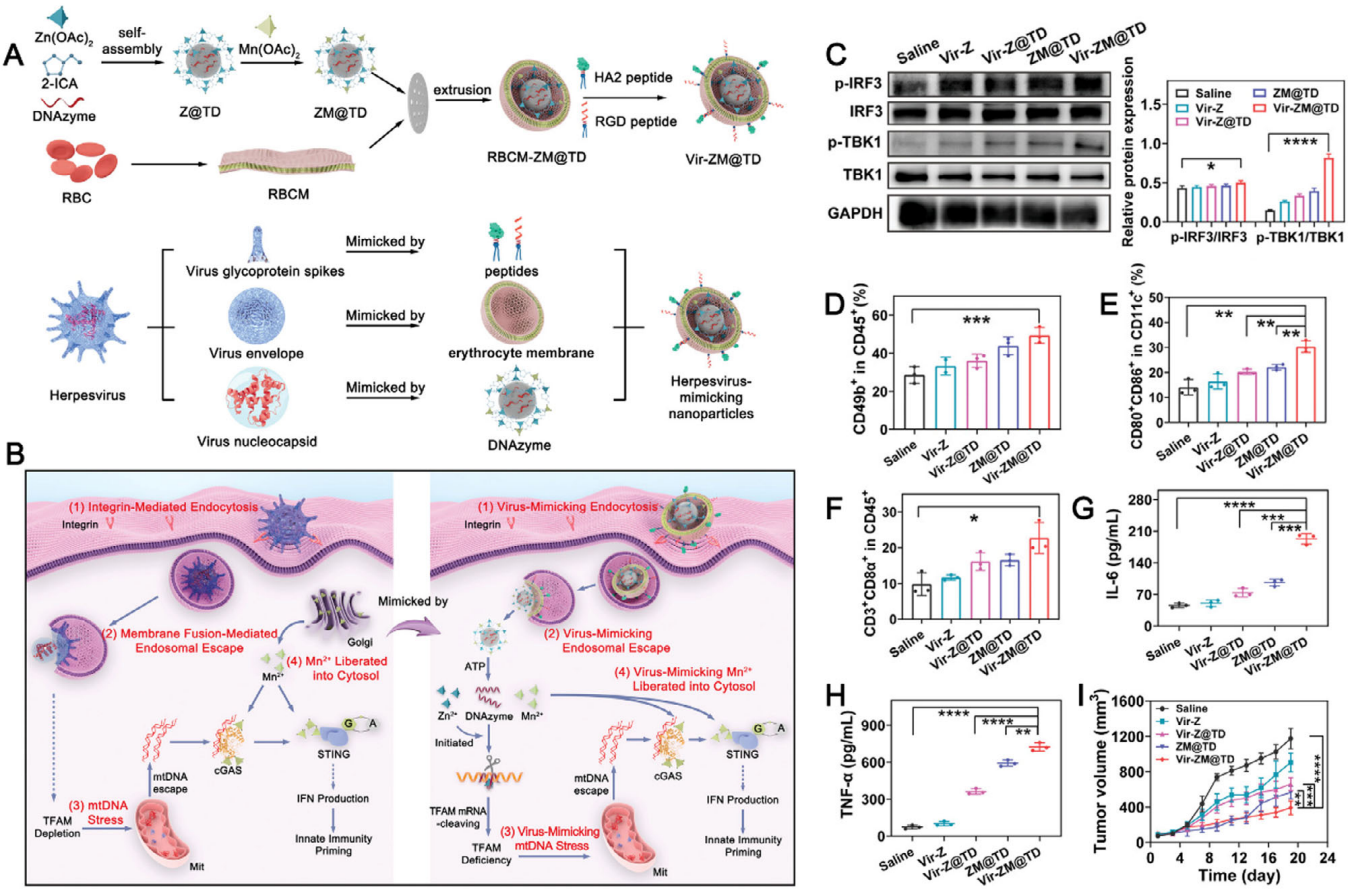
(A) The synthetic process of Vir‐ZM@TD and the illustrative mechanism harnessed by Vir‐ZM@TD to mimic herpes virus. (B) The schematic illustration of the immunotherapeutic mechanism harnessed by Vir‐ZM@TD to activate the cGAS‐STING pathway. (C) Immunoblotting of IRF3, TBK1, and phosphorylation of the corresponding proteins extracted from tumor tissues after different treatments and quantitative analysis, including saline, Vir‐Z, Vir‐Z@TD, ZM@TD, and Vir‐ZM@TD treatments. (D–F) Flow cytometry analysis of different immune cells residing in tumor tissues after different treatments. Serum levels of (G) IL‐6 and (H) TNF‐α after different treatments. (I) Antitumor growth curves of the primary tumor in different treatment groups. Reprinted with permission.^[^ ^37^ ^]^ Copyright 2022, Wiley.

Mn^2+^ can also be doped into other MOF structures. For example, Zhao and coworkers developed a herpes virus‐mimicking biomimetic system (Vir‐ZM@TD) that is composed of DNAzyme‐loaded Mn^2+^‐doped ZIF‐90 NPs as the virus nucleocapsid, erythrocyte membrane as the viral envelope, and RGD and HA2 as the glycoprotein spikes (Figure 4).^[^ ^37^ ^]^ This sophisticated biomimetic system elicited strong and persistent antitumor immune responses mainly by initiating herpes virus‐mimicking mtDNA stress to activate the cGAS‐STING pathways. Mechanistically, RGD enabled specific tumor targeting, while HA2 led to membrane fusion‐mediated endosomal escape like herpes virus. Subsequently, Zn^2+^, DNAzyme, and Mn^2+^ disintegrated from the biomimetic system, which triggered cleavage of mtDNA‐binding protein TFAM (transcription factor A, mitochondrial) and mtDNA stress, leading to the influx of mtDNA into the cytosol. With the aid of Mn^2+^, mtDNA activated the cGAS‐STING pathways to achieve significant antitumor immune responses. The intratumoral immune stimulation was demonstrated by the elevated frequency of CD49b^+^ NK cells, CD80^+^CD86^+^ DCs, CD3^+^CD8^+^ T cells, as well as enhanced levels of inflammatory cytokines (IL‐6 and TNF‐α). Ultimately, effective tumor suppression in primary and distant 4T1 tumors was achieved by Vir‐ZM@TD. In a recent study, Mn^2+^‐based immunostimulatory MOFs were constructed to initiate cancer metalloimmunotherapy by simply mixing MnCl_2_ and sodium salt of squaric acid (SA) in an aqueous alcohol solution, which were demonstrated to activate cGAS‐STING signaling pathway in DCs.^[^ ^38^ ^]^

In addition, free metal ions could also be directly loaded into typical organic nanomaterials, such as liposomes, PLGA, and polyamidoamine.^[^ ^39^ ^]^ Free Mn^2+^ was reported to be loaded into a phospholipid bilayer shell for cGAS/STING signaling pathway activation and ROS‐induced ICD in a 4T1 tumor model.^[^ ^40^ ^]^

To sum up, among all the listed nanomaterials, MONPs seem to be the most frequently used one to serve as Mn nanoreservoir because of their stability in circulation and redox reaction in the TME. However, it is still worth exploring other nanosystems, such as the organic‐inorganic hybrid nanomaterials, since they possess better TME responsiveness, thus releasing Mn more rapidly in the TME.

### 2.2 Iron (Fe)

Fe is the most abundant transitional metal in the human body. Due to its constant valence switch between Fe^2+^ and Fe^3+^, Fe plays major roles in a lot of biological activities, including enzymatic functions, oxygen transport, ATP synthesis, and redox balance. In recent years, elevated Fe level was constantly found to be associated with enhanced anticancer immunity, including inducing immunogenic cell death (ICD), and remodeling immunosuppressive TME by repolarizing TAMs into M1 phenotype. In this part, we summarized the major signaling pathways involving Fe in antitumor immune responses and introduced Fe‐involved nanomaterials that can assist in delivering Fe element into the tumor site precisely without inducing systemic free metal toxicity.

#### 2.2.1 Immune‐related pathways

##### ICD pathways

In the acidic TME, the introduction of abundant Fe can catalyze the production of ROS via typical Fenton reaction, thereby leading to ICD under excessive H_2_O_2_ in situ. Fe_3_O_4_ NPs containing both Fe^2+^ and Fe^3+^ are the most popular catalysts for Fenton reactions. Fe^2+^ holds stronger catalytic activity than Fe^3+^, thereby inducing stronger Fenton reaction and producing larger amount of ROS.^[^ ^10a^ ^]^ From another perspective, Fe^3+^ can function as GPx and CAT‐like enzymes to degrade GSH and generate O_2_, thus preventing the degradation of ROS by GSH and alleviating intratumoral hypoxia simultaneously. Other than Fe_3_O_4_ NPs, various iron‐based nanocomplexes have also been reported to induce effective ICD effect via Fenton reaction and GSH depletion.^[^ ^41^ ^]^

##### TAM repolarization

Excessive intracellular ROS can not only induce ICD but also repolarize pro‐tumoral M2 TAMs into the tumoricidal M1 phenotype.^[^ ^23^ ^]^ Fe_3_O_4_ which contains both Fe^2+^ with high catalytic activity and Fe^3+^ with low catalytic activity, has been applied in numerous studies to catalyze the production of ROS for TAM polarization.^[^ ^41^, ^42^ ^]^ Based on the interplay between ROS, NF‐κB pathway activation, and TAM polarization, some researchers ascribed the underlying mechanism of Fe_3_O_4_‐induced TAM re‐polarization to the ROS‐TLR/NF‐κB signaling pathway.

Besides NF‐κB, IRF5, which is one of the classical transcription factors, was found to be associated with Fe‐mediated M1 TAM polarization signaling pathways by producing IL‐23, and thereby activating CD8^+^ T cells and facilitating the proliferation of memory T cells.^[^ ^43^ ^]^

**FIGURE 5:**
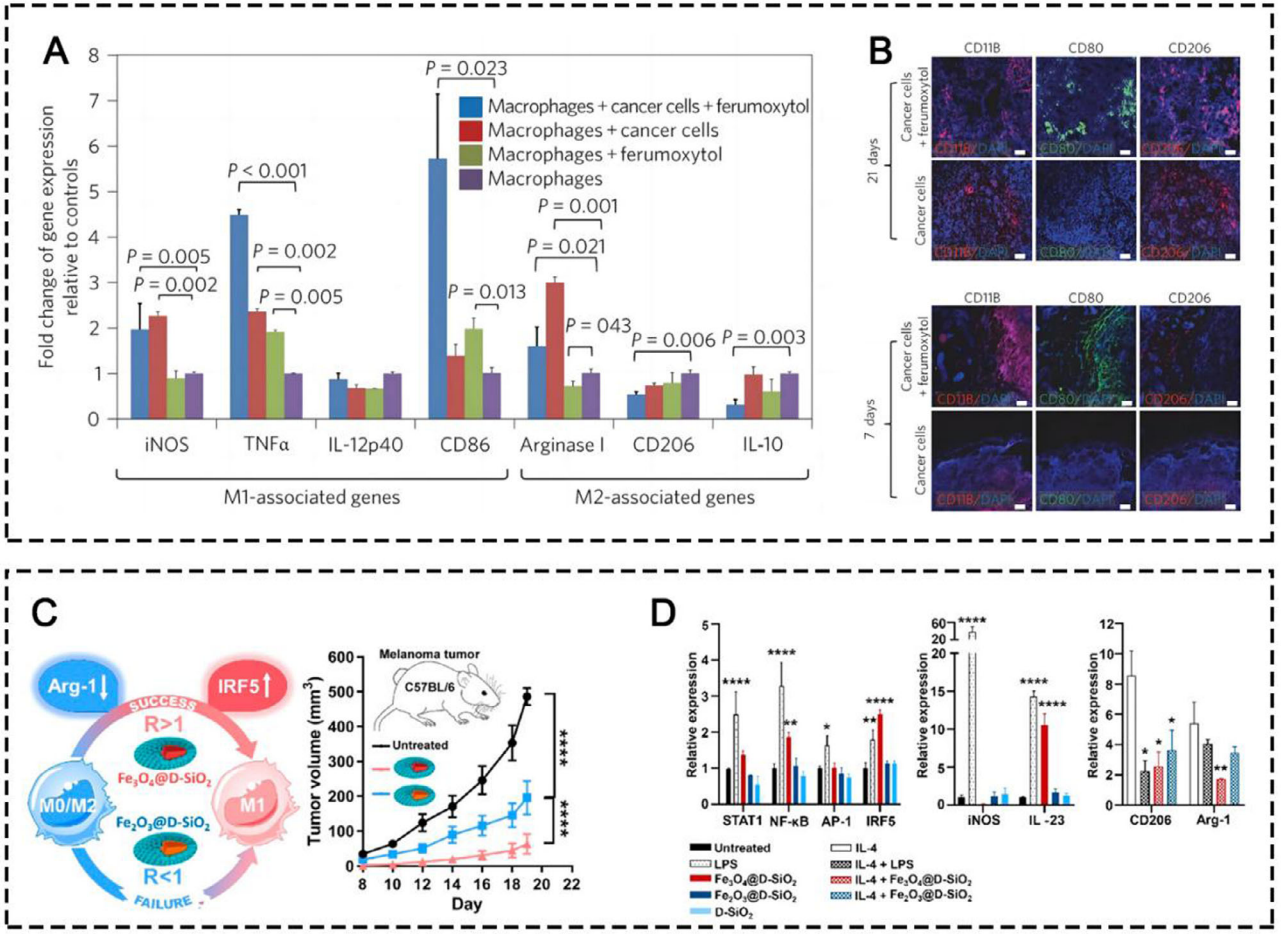
(A) Quantitative RT‐PCR (qRT‐PCR) of M1/M2‐associated gene expression measured in cocultures of cancer cells, macrophages, and ferumoxytol. (B) Representative immunofluorescence staining for CD11b (red), CD206 (red), and CD80 (green) of MMTV‐PyMT tumor sections obtained at (A) 7 days and (B) 21 days after implantation of 2.3×106 cancer cells with or without ferumoxytol (2.73 mg Fe/mL). Scale bar, 100 and 50 µm. Reprinted with permission. ^[^ ^11c^ ^]^ Copyright 2016, Springer Nature. (C) Illustrative mechanisms of TAM polarization induced by iron oxide NPs. (D) Signaling pathway study of M0 macrophage (RAW264.7) treated with LPS, IONP@D‐SiO_2_, and D‐SiO_2_ as measured by qRT‐PCR. Reprinted with permission.^[^ ^11d^ ^]^ Copyright 2019, American Chemical Society.

In 2016, Zanganeh et al. reported that the Food and Drug Administration (FDA)‐approved iron supplement, ferumoxytol, was able to induce the repolarization of M2‐like TAMs into M1‐like TAMs, leading to an effective tumoricidal effect, which is dependent on the production of ROS induced by Fe_3_O_4_ in the TME.^[^ ^11c^ ^]^ The mechanism was verified by increased expression levels of M1 biomarkers CD80 and TNF‐α, and decreased expression levels of M2 biomarkers CD206 and IL‐10 (Figure 5A,B). Another study in 2019 explored the underlying mechanism (Figure 5C,D).^[^ ^11d^ ^]^ They studied four classic M1 signaling pathways, including STAT1, NF‐κB, AP‐1, and IRF5. The results of qRT‐PCR and western blotting demonstrated that Fe_3_O_4_ activated both NF‐κB and IRF5, while only activating the expression level of IL‐23 downstream of IRF5. The downstream of the NF‐κB signaling pathway, iNOS, displayed negligible expression, possibly because iron could attenuate iNOS expression. Since then, several preclinical studies evaluated the mechanism of Fe‐induced TAM polarization and corroborated that IRF5 signaling pathways played a pivotal role in this process.^[^ ^24^, ^44^ ^]^

#### 2.2.2 Fe‐containing nanomaterials

Similar to Mn, excessive Fe content in human body causes severe side effects and metal toxicity, including vasoconstriction, hypertension, and oxidative organ damage. Thus, it is essential to utilize nanomaterials as a metal delivery platform. Hence, we listed the most common categories of Fe‐containing nanomaterials that were reported to elicit metalloimmunotherapy.

##### Inorganic nanomaterials

**FIGURE 6:**
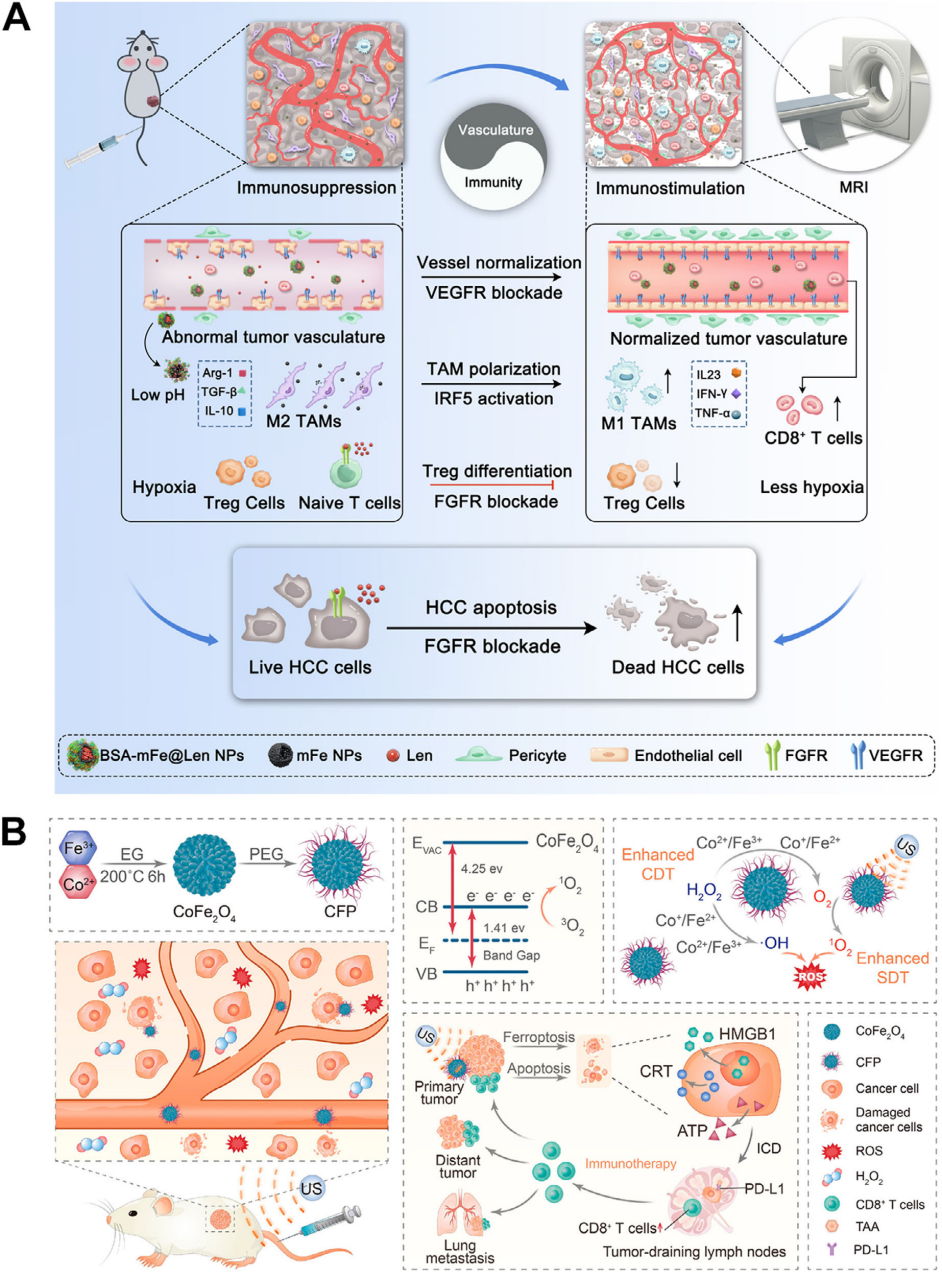
(A) Illustrative mechanism of BSA‐mFe@Len NPs modulating the immunovascular crosstalk for combating HCC. Reprinted with permission.^[^ ^44c^ ^]^ Copyright 2022, Elsevier. (B) Synthetic procedure of CFP and its mechanism for augmented sonodynamic and chemodynamic combination therapy with elicitation of robust immune response. Reprinted with permission.^[^ ^47^ ^]^ Copyright 2021, American Chemical Society.

Iron oxide NPs (IONPs), are one of the most common inorganic nanomaterials applied in nanomedicine for cancer theranostics, which enable not only multimodality imaging, effective drug delivery, photothermal responsiveness, and magnetothermal responsiveness, but also possess intrinsic antitumor immunomodulatory effects.^[^ ^45^ ^]^ IONPs that contain both Fe^2+^ and Fe^3+^ induce intrinsic metalloimmunotherapy mainly through three mechanisms: i. Fe^2+^ catalyzes the Fenton reaction to produce abundant ROS to induce CDT and concurrent ICD for evoking innate and adaptive immune systems to destroy cancer cells; ii. Fe^3+^ ions deplete excessive GSH to prevent ROS elimination for amplifying the ICD effect; iii. Fe ions directly induce TAM polarization to obtain the antitumor M1 phenotype. Recently, ferumoxytol and other IONPs with different sizes and structures have been employed to inhibit the growth of a myriad of solid tumors based on TAM polarization,^[^ ^11^, ^24^, ^41^, ^42^, ^44^, ^46^ ^]^ via IRF5‐IL23 signaling pathway or NF‐kB signaling pathway activation. Combination interventions that are intended to assist in the therapeutic efficacy of IONPs, such as chemotherapy, physiochemical therapies, TME modifiers, and other immunotherapeutic agents have also been applied. Additionally, copolymer materials, such as PAA, dihydro‐5‐azacytidine (DHAC), silica (SiO_2_), bovine serum albumin (BSA), PLGA, and organosilica have been exploited to coat naked IONPs to prolong their circulation time, enhance TME responsiveness, and provide active groups to conjugate other small drug molecules for synergistic therapy. Furthermore, biomimetic membranes, like natural leukocyte membranes, MDSC membranes, macrophage membranes, platelet (PLT) membranes, and genetically engineered cell membranes have also been applied to camouflage naked IONPs to avoid immune clearance, prolong NP circulation time, and actively target tumor cells or the inflammatory TME via some tumor‐homing molecules anchored on the carrier membranes. Very recently, our research team synthesized mesoporous Fe_3_O_4_ NPs (mFe NPs) loaded with Lenvatinib (Len), which were further coated with a BSA corona for combating liver cancer (Figure 6A).^[^ ^44c^ ^]^ In our research, the mFe NPs not only served as Len carrier and *T*_2_‐weighted MRI agent but also exerted anticancer immunity by TAM polarization via IRF5‐IL23 signaling pathway. Simultaneously, low‐dose Len delivered by the NPs induced multiple regulatory functions in the TME, including direct triggering of tumor apoptosis, vessel normalization, cytotoxic T‐lymphocyte recruitment, and regulatory T‐cell elimination. In a concerted effort, the BSA‐mFe@Len NPs effectively inhibited the Hepa1‐6 tumor growth and significantly prolonged mouse survival.

Interestingly, metal oxides not only can be present in monometallic forms, metal oxides with poly‐metallic forms can also exist and possess strong enzyme‐like activities. For instance, Fu and co‐workers synthesized PEGylated CoFe_2_O_4_ nanoflowers (CFP) with multiple multivalent elements (Co^2+^/Co^3+^, Fe^2+^/Fe^3+^) which exhibited strong CAT‐like and POD‐like activities, leading to efficient ICD effect. The combination with ICBs led to suppression of primary and distant 4T1 tumor growth (Figure 6B).^[^ ^47^ ^]^ In another study, iron tungsten oxide (FeWO_x_)‐based nanosheets were synthesized as a triple‐modality therapy for treating 4T1 tumors.^[^ ^48^ ^]^ The FeWO_x_ nanosheets were demonstrated to possess Fe^2+^, Fe^3+^, and W^6+^. Fe^2+^ released from the nanosheets catalyzed the Fenton reaction to produce ROS for ICD induction, while Fe^3+^ and W^6+^ with high valence states depleted excessive GSH in the TME to prevent ROS elimination. Moreover, this study exploited near‐infrared (NIR)‐responsiveness of the FeWO_x_ to induce both PDT and PTT, further amplifying the ICD process and antitumor immune responses in combination with anti‐PD‐L1 antibody treatment.

Lately, inorganic 2D Fe‐based nanosheets (FeNSs) were constructed for metalloimmunotherapy.^[^ ^49^ ^]^ The authors reported that the basic structure unit of FeNSs was speculated to be Fe_3_(salicylate)_3_(OH)_3_(2H_2_O)_3_·14H_2_O. In this study, The FeNSs were used for TAM polarization, PTT, PDT, and immune‐activated chemotherapy. What's inspiring in this study was that after TAM polarizing into M1 phenotype by Fe ions, the chemotherapeutic prodrug, non‐toxic AQ4N, can be converted into toxic AQ4 by iNOS overexpressed by M1 TAMs. Therefore, this study achieved not only TAM‐based immunotherapy but also immune‐activated chemotherapy, which was further amplified by PTT and PDT due to the photo‐responsiveness of the FeNSs, leading to efficient 4T1 tumor suppression.

In a recent study, both IONPs and MONPs were integrated into one nanoplatform to exert multiple immunomodulatory effects of both Fe and Mn ions. Zhao et al. synthesized yolk–shell nanohybrids that were composed of Fe_3_O_4_ core, carbon/MnO_2_ shell, and low‐toxic cationic polymer CD‐PGEA (FCMP), which achieved efficient ICD effect via the Fenton and Fenton‐like reaction catalyzed by both Fe^2+^ and Mn^2+^ and immune activation effects mediated by Fe ion‐induced IRF5‐IL23 signaling pathway and Mn ion‐induced cGAS‐STING signaling pathway for treating 4T1 tumor‐bearing mice.^[^ ^24d^ ^]^

##### Organic–inorganic hybrid nanomaterials

It is worth noting that metal ions with low catalytic activities can be incorporated with nanomaterials with high reducibility (such as tannic acid), therefore, metal elements can remain stable in circulation and are transformed into ionic forms in a lower valence state with higher catalytic activities by reducing agents and endogenous stimuli.

Recently, nanoscale MOFs (nMOFs), as a type of hybrid crystalline materials built by metal ion cores and organic ligands, have shown great potential in nanomedicines due to their high molecular loading/releasing capacities, structural/chemical diversities, and intrinsic biocompatibility/degradability.^[^ ^50^ ^]^

**FIGURE 7:**
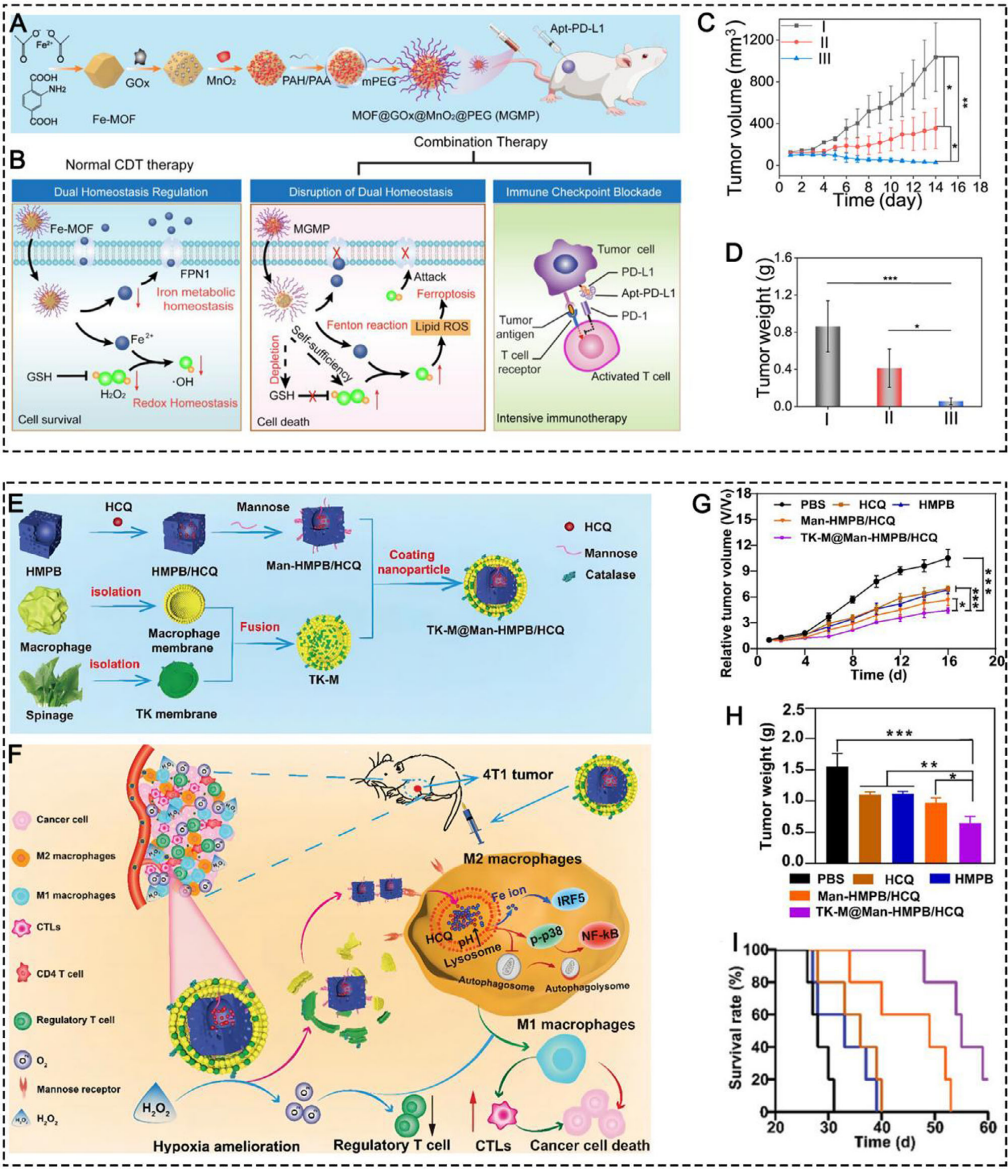
Schematic illustration of (A) the synthesis of MGMP and (B) its therapeutic mechanism of ferroptosis‐based immunotherapy. (C) The antitumor growth curve and tumor weight in different treatment groups, including (I) PBS, (II) MGMP+R.S. Apt‐PD‐L1, and (III) MGMP+Apt‐PD‐L1. Reprinted with permission.^[^ ^51^ ^]^ Copyright 2022, Elsevier. Schematic illustration of (E) the fabrication of TK‐M@Man‐HMPB/HCQ and (F) its therapeutic mechanism of TAM‐based immunotherapy. (G) The antitumor growth curve, (H) tumor weight, and (I) mouse survival observation in different treatment groups, including PBS, HCQ, HMPB, Man‐HMPB/HCQ, and TK‐M@Man‐HMPB/HCQ. Reprinted with permission.^[^ ^44b^ ^]^ Copyright 2022, Wiley.

Zhang and co‐workers reported a kind of Fe‐MOF based on the coordination between Fe^2+^ and 2‐aminoterephthalic acid (Figure 7A–D).^[^ ^51^ ^]^ The Fe‐MOF nanocomposites that were complexed with MnO_2_, GO_x_, and PEG (MOF@GOx@MnO_2_@PEG (MGMP)), achieved successful ferroptosis‐induced immunotherapy in 4T1 tumor‐bearing mice when in cooperation with anti‐PD‐L1 antibody. Prussian blue (PB) NPs, which are composed of Fe^2+^ and Fe^3+^ to form ferric ferrocyanide, are also a kind of nMOFs.^[^ ^52^ ^]^ In 2022, Hou et al. constructed a mannose‐decorated hollow mesoporous PB nanoplatform with hydroxychloroquine (HCQ) encapsulation (Man‐HMPB/HCQ) for TAM targeting and polarization (Figure 7E–I).^[^ ^44b^ ^]^ In this nanoplatform, both Fe ions and HCQ contributed to the TAM polarization. To avoid reticuloendothelial system (RES) uptake, enhance tumor accumulation, and alleviate tumor hypoxia, macrophage and thylakoid hybrid membranes were coated on the NP surface. The Man‐HMPB/HCQ NPs were demonstrated to significantly increase the M1/M2 ratio of TAMs and CTL proportion and decrease Tregs to combat 4T1 tumor growth and enhance mouse survival time.

Besides typical MOF and MPN structures, metal–organic coordination copolymers can be established through the coordination between metal ions with other organic ligands, such as polydopamine (PDA), covalent organic framework (COF), QDs, and even small drug molecules.

Sun et al. developed coordination copolymers by Fe^3+^, 5‐azosalicylic (5‐ASA), and a hypoxia‐sensitive bond azobenzene (N═N) group (PEGylated FeNCPs).^[^ ^53^ ^]^ This nano drug exploited the GPx‐like activity of Fe^3+^ and the POD‐like activity of Fe^2+^ for GSH depletion and ROS production. In addition, the Fe^3+^ was also exploited to polarize immunosuppressive M2 TAMs into antitumoral M1 mode. In response to intratumoral hypoxia, 5‐ASA was released to inhibit the activity of cyclooxygenase‐2, which contributed to ROS production for ICD effect. The multiple immunomodulatory effects induced by the PEGylated FeNCPs led to significant tumor inhibition in the 4T1 tumor model.

PDA is a type of well‐known organic material that possesses strong chelation ability with metal ions.^[^ ^54^ ^]^ Fe^3+^ has been reported by several studies to chelate PDA for TAM repolarization.^[^ ^44^, ^55^ ^]^ With the aid of PDA‐induced PTT, TAAs were released from dying tumor cells to potentiate M1 TAMs to become APCs, thereby presenting antigens to T cells for activating the adaptive immune system in both CT26 and 4T1 tumor‐bearing mice.

COF is an emerging class of novel nanomaterials that is made from light elements through reversible covalent bonds.^[^ ^56^ ^]^ Wang et al. coordinated Fe^3+^ with a porphyrin‐based COF structure with sonodynamic performance to achieve both CDT and SDT, which synergistically led to ICD, resulting in efficient antitumor therapeutic effect in 4T1 tumor models.^[^ ^57^ ^]^

Sulfur quantum dots (S dots) which contain abundant oxygen‐containing functional groups (hydroxyl groups, carboxyl groups, and sulfonic groups) on their surface, can absorb metal ions by forming a coordination bond with oxygen atoms on the surface.^[^ ^58^ ^]^ Sang et al. constructed TAM‐targeting peptides‐modified S dots to adsorb endogenous Fe^3+^ ions (denoted as SPF).^[^ ^59^ ^]^ The SPF served as endogenous Fe^3+^ nanoreservoir with ultrasmall size (6.5 nm in hydrodynamic size and 3.2 nm under TEM), which penetrated deeply into the tumor center and released Fe^3+^ inside TAMs due to the gradual etching of S dots in response to excessive H_2_O_2_. The Fe^3+^ caused ROS production which promoted TAM polarization into the M1 mode, leading to effective inhibition of 4T1 breast tumor growth. Exploiting endogenous Fe ions for metalloimmunotherapy is ingenious since it avoids the systemic metal toxicity caused by exogenous metal ions and re‐utilizes endogenous metabolic wastes as therapeutic agents. Lately, Liu et al. applied a virus‐like hijacking strategy to develop intracellular Fe^2+^ coordinated bisphosphonates for TAM polarization with the aid of vascular disrupting agents such asCA4P and R300.^[^ ^60^ ^]^ This study was based on the efferocytosis of erythrocytes by TAMs. Systemically injected VDA disrupted tumoral vasculature and caused erythrocyte leakage. TAMs phagocytosed these erythrocytes and produced Fe^2+^ from engulfed hemoglobin by heme oxygenase‐1. Fe^2+^ self‐assembled with systemically injected bisphosphonates into the final Fe^2+^ coordinated bisphosphonates to cause ROS production and thereby activate TAMs for antitumor immune responses as determined in a CT26 tumor model.

Metal ions can also coordinate with some small drug molecules. For example, Fe^3+^ was coordinated with methotrexate (MTX) to self‐assemble into nanoscale coordination polymers which induced immunogenic tumor ferroptosis as determined by a research team in 2022.^[^ ^51^ ^]^

Hou and coworkers immobilized Fe^3+^ on the surface of carbon dots (CDs) for ROS generation and ICD induction.^[^ ^61^ ^]^ Fe ions were also doped into nanoconjugates consisting of GOx and PDMA and then in situ reduced to Fe^0^ by sodium borohydride.^[^ ^62^ ^]^ Fe^0^ in the GOx‐PDMA‐Fe^0^ was ionized into Fe^2+^ at low pH with the help of gluconic acid catalyzed by GOx, and then initiated Fenton reaction to induce CDT and concurrent ICD which was amplified by GOx‐induced starvation therapy. In another study, both Fe^2+^ and Mn^2+^, in a form of metal silicate were coated on the surface of dendritic mesoporous SiO_2_ nanospheres which also incorporated TGF‐β inhibitor (TI).^[^ ^63^ ^]^ In this study, Fe^2+^ and Mn^2+^ possessed POD and CAT‐like activities to produce O_2_ and ROS in the TME in response to the intratumoral excessive H_2_O_2_, leading to hypoxia alleviation, ICD, and TAM polarization in synergy with TI.

Overall, regarding all Fe‐related nanomaterials, IONPs seem to be the most promising candidate for entering clinical translation in metalloimmunotherapy, since the versatility, stability, and biosafety of IONPs are well‐explored and guaranteed. Other Fe‐containing inorganic nanomaterials and Fe ion‐coordinated organic co‐polymers lack systematic studies, thus entailing further evaluation in terms of pharmacokinetics, in vivo toxicity, antitumor efficacy, and so on. It is worth noting that ferric iron seems to be pivotal in inducing tumor metalloimmunotherapy according to current research.

### 2.3 Copper (Cu)

Cu is a redox active transition metal, an essential nutrient, and a co‐factor of several metalloenzymes with central roles in various cellular processes.^[^ ^8^ ^]^ In cancer, enhanced Cu levels promote tumor growth and metastasis. High levels of Cu in the blood and tumor tissues of cancer patients have been reported to be correlated for a wide variety of malignancies, which was already summarized.^[^ ^8^, ^64^ ^]^ Thus, Cu chelating agents were applied to interact with endogenous Cu and sequester Cu from the tumor sites. However, Cu chelating agents were reported to decrease antitumor immunity and dampen the activities of various antitumoral immune cells. Furthermore, in contrast to Cu chelating agents, Cu ionophores were used to kill cancer cells by causing high cytotoxicity, since the switch between Cu^+^ and Cu^2+^ leads to oxidative reactions and the production of abundant cytotoxic free radicals. Especially, recent research found that enhanced intracellular Cu level causes a new form of cell death, cuproptosis, which may be helpful in combating malignant tumor growth.^[^ ^65^ ^]^ In the field of metalloimmunotherapy, various findings support the idea that elevated Cu level in tumor site causes immune stimulation, including inducing ICD and repolarizing TAMs. In this part, we summarized the major signaling pathways involving Cu in antitumor immune responses and introduced Cu‐containing nanomaterials that can assist in delivering Cu element into the tumor site precisely without inducing systemic toxicity of free metal.

#### 2.3.1 Immune‐related pathways

##### ICD pathways

Recently, Cu‐based Fenton‐like reagents have attracted considerable interest because of their superior catalytic activity in a broad pH range.^[^ ^66^ ^]^ It is worth noting that Cu^+^/Cu^2+^ has a higher catalytic efficiency than the most typical Fenton reaction catalyst, Fe^2+^, and the conversion from Cu^2+^ to Cu^+^ is faster than that from Fe^3+^ to Fe^2+^.^[^ ^10a^ ^]^ Based on the intensive Fenton‐like reaction catalyzed by Cu ion, excessive ROS in tumor cells disables mitochondrial function and attacks nuclear DNA, thereby inducing ICD. Abundant DAMPs and tumor antigens released from tumor cells promote DC maturation, leading to the activation of the innate and adaptive immune systems and triggering antitumor immunity.

##### TAM repolarization

The excessive intracellular ROS induced by Cu can not only induce the ICD but also repolarize pro‐tumoral M2 TAMs into the tumoricidal M1 phenotype. Cu‐based nanomaterials have been well documented to polarize TAMs, which was associated with ROS production like Fe ions, thus it is reasonable to speculate that the downstream of ROS, NF‐κB, may play a part in Cu‐mediated TAM repolarization process. In addition, Cu‐containing nanomaterials also possess CAT‐like activities and can produce O_2_ in the TME, leading to hypoxia alleviation and thereby TAM repolarization. As an example, Cu^2+^ was applied to coordinate with polypyrrole to develop a nanozyme which possesses CAT activity to produce O_2_ in the TME for hypoxia alleviation, thus re‐educating M2 TAMs to polarize into the M1 mode.^[^ ^67^ ^]^ In addition, another inorganic compound, ultra‐small Cu_2−x_Se NP^[^ ^68^ ^]^, was reported to induce TAM polarization and inhibit the growth of melanoma tumors prophylactically, primarily, and recurrently through the IRF5 signaling pathway.^[^ ^69^ ^]^

##### Relationship between cuproptosis and immune stimulatory pathways

Cuproptosis refers to a newly reported programmed cell death caused by overload of Cu^2+^. In short, the overload of Cu^2+^ leads to aggregation of lipoylated proteins and loss of Fe‐S clusters, thereby promoting proteotoxic stress and culminating in cell death.^[^ ^64^ ^]^ Presently, research into cuproptosis and the relationship between cuprotosis and immune compartment is still in its infancy. Even though numerous studies revealed that cuproptosis was correlated with immunostimulatory cell infiltration and immunosuppressive cell depletion in the TME, including DCs, CD4^+^, or CD8^+^ T cells, Tregs, and TAMs, the crossroad of specific copper signaling pathway and immune activation remains to be explored.^[^ ^68^ ^]^ Recently, Li and colleagues revealed that similar to ferroptosis and pyroptosis, cuproptosis was immunogenic, which could induce dying cells to release abundant DAMPs and then repolarize M2 TAMs into M1 phenotype and promote DC maturation, ultimately leading to T cell infiltration.^[^ ^70^ ^]^ Zhang and coworkers also revealed cuproptosis induced intensive immunogenic cell death as demonstrated by release of a large number of DAMPs and initiation of innate and adaptive antitumor immunity.^[^ ^71^ ^]^ However, in some studies, copper signaling pathway was reported to increase PD‐L1 expression on tumor cells, thus enabling tumor immune evasion, which is contradictory to cuproptosis‐induced antitumor immunity. To intercept tumor immune evasion caused by PD‐L1 expression, Zhang's group co‐administered JQ1 to suppress bromodomain‐containing protein 4 (BRD4) and exploited the CAT‐like activity of Cu^2+^ to produce O_2_ to ameliorate cuproptosis‐induced PD‐L1 overexpression. Anyway, the understanding of cuproptosis and the related antitumor immune responses is still fragmented and remains to be further explored.

#### 2.3.2 Cu‐containing nanomaterials

Maintenance of Cu homeostasis is essential since free Cu ions are highly toxic and require a tightly regulated Cu transport and distribution system. Nanomaterials provide a solution to this problem.

##### Inorganic nanomaterials

**FIGURE 8:**
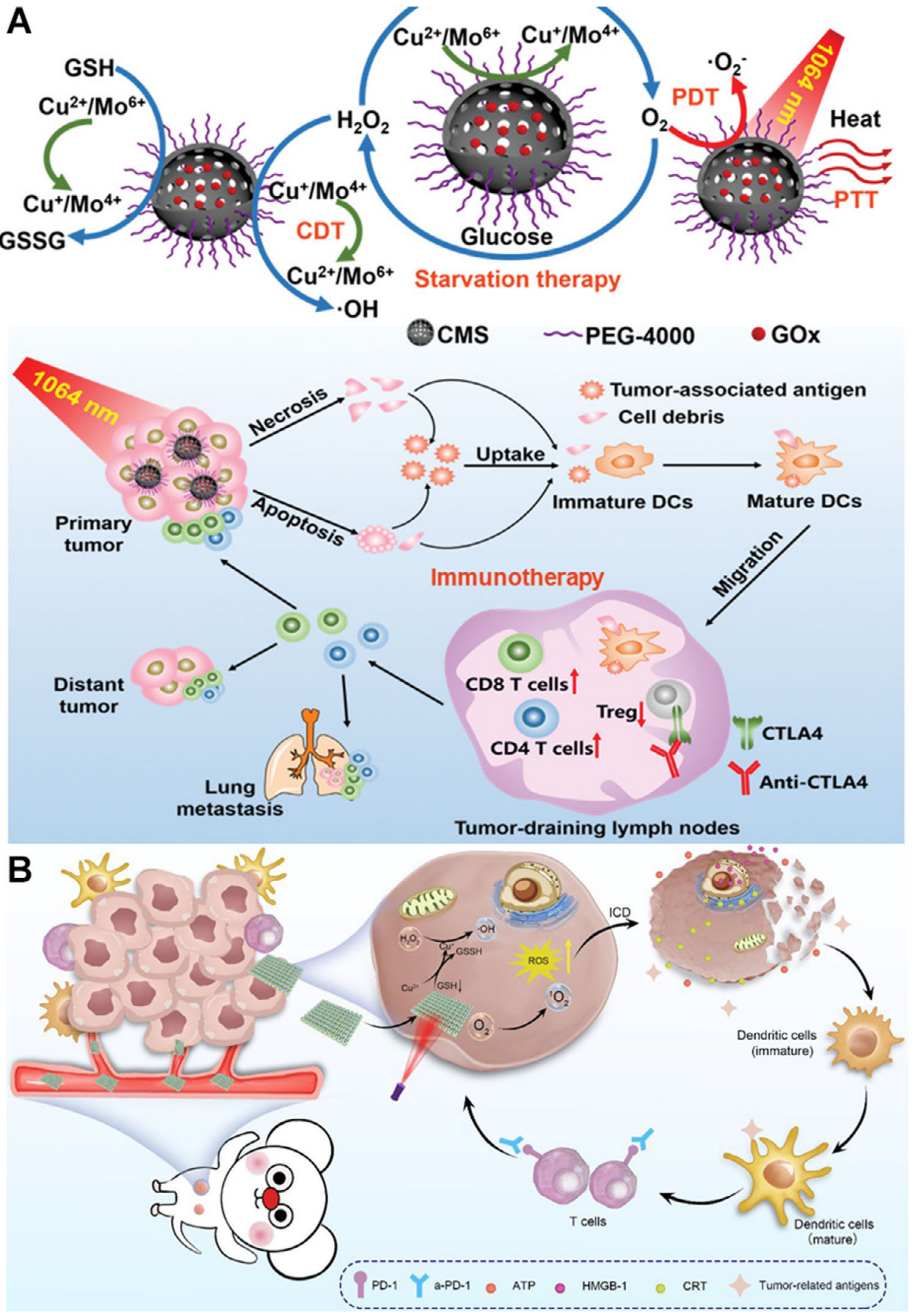
(A) Schematic illustration of synthesis and mechanism of PEGylated CMS@GOx for PTT, PDT, CDT, and starvation therapy. Reprinted with permission.^[^ ^72^ ^]^ Copyright 2019, Wiley. (B) Schematic Illustration of 3D Cu@COF‐TATB for Cancer Immunotherapy via PDT‐ and CDT‐triggered ICD. Reprinted with permission.^[^ ^73^ ^]^ Copyright 2012, American Chemical Society.

In addition to iron/manganese oxides, cuprous oxide NPs have also been explored to induce ROS‐induced ICD through catalyzing Fenton‐like reactions or disrupting mitochondria function. Recently, Chang et al. developed a multifunctional cascade bioreactor based on hollow structured Cu_2_MoS_4_ (CMS) harboring multivalent elements (Cu^+^/^2+^ and Mo^4+^/^6+^) which loaded glucose oxidase (GOx) for cancer CDT, starvation therapy, and immunotherapy (Figure 8A). Cu and Mo ions with multiple valences possess POD, CAT and GPx enzyme‐like activities, thus the CMS can trigger the production of ROS, O_2_, and the depletion of redundant GSH in the TME, leading to efficient CDT accompanied by ICD effect.^[^ ^72^ ^]^ In this study, the authors also utilized the photothermal responsiveness of CMS for PTT and GOx for simultaneous starvation therapy. More importantly, they found that the CMS induced vaccine‐like immune activities, which promoted DC maturation and T‐cell activation. In combination with anti‐CTLA4 antibodies, the multifunctional nanocomposites significantly inhibited the growth of the primary and distant cervical tumors and prolonged the survival of cervical tumor‐bearing mice compared with the other groups.

##### Organic‐inorganic hybrid nanomaterials

Similar to Fe/Mn coordinated organic nanomaterials, Cu ion can also form organic‐inorganic hybrid nanomaterials with certain organic ligands. Porphyrin‐containing 3D COF (3D COF‐TATB) was used to coordinate with Cu^2+^ to deplete GSH, produce ROS, and thereby induce ICD to activate a strong immune response for attacking 4T1 tumor when combined with anti‐PD‐1 antibody (Figure 8B).^[^ ^73^ ^]^ In another study, multienzyme‐mimicking COFs (COF‐909‐Cu, COF‐909‐Fe, and COF‐909‐Ni) were fabricated via a coordination bond, leading to intensive antitumor immunity when combined with ICB.^[^ ^74^ ^]^ Among the above three metal‐doped COF structures, COF‐909‐Cu robustly induced GSDME‐dependent pyroptosis which is a type of ICD and can initiate intense antitumor immunity. Zeng et al. developed a strategy to synthesize polypyrrole (PPy) by replacing FeCl_3_ with CuCl_2_ as an oxidizing catalyst via a facile and one‐step green synthesis. The synthesized Cu‐doped PPy nanozymes, containing mixed metal valance states (Cu^+^/Cu^2+^), were demonstrated to possess trienzyme‐like activities, including CAT, POD, and GPx, to specifically promote O_2_ and ·OH induction as well as GSH reduction in TME, causing irreversible oxidative stress damage to tumor cells and inducing macrophage polarization.^[^ ^67^ ^]^ Cu^2+^ was also reported to be immobilized on upconversion NPs and dendritic mesoporous organosilica NPs to have POD‐like activities.^[^ ^75^ ^]^

### 2.4 Calcium (Ca)

Ca^2+^ is the most well‐studied and widely accepted ionic second messenger in regulating various immune responses. The Ca^2+^ influx is particularly important at each step of cell cycle progression and the proliferation of the immune cells. Slight change to Ca^2+^ homeostasis can greatly disturb normal physiological conditions. In the field of antitumor immunity, Ca^2+^ concentration was usually found to be positively related with the intensity of antitumor immune responses. In this part, we summarized the major signaling pathways involving Ca in antitumor immune responses and introduced Ca‐containing nanomaterials that can assist in delivering Ca element into the tumor site precisely without inducing systemic free metal toxicity.

#### 2.4.1 Immune‐related pathways

##### ICD pathways

**FIGURE 9:**
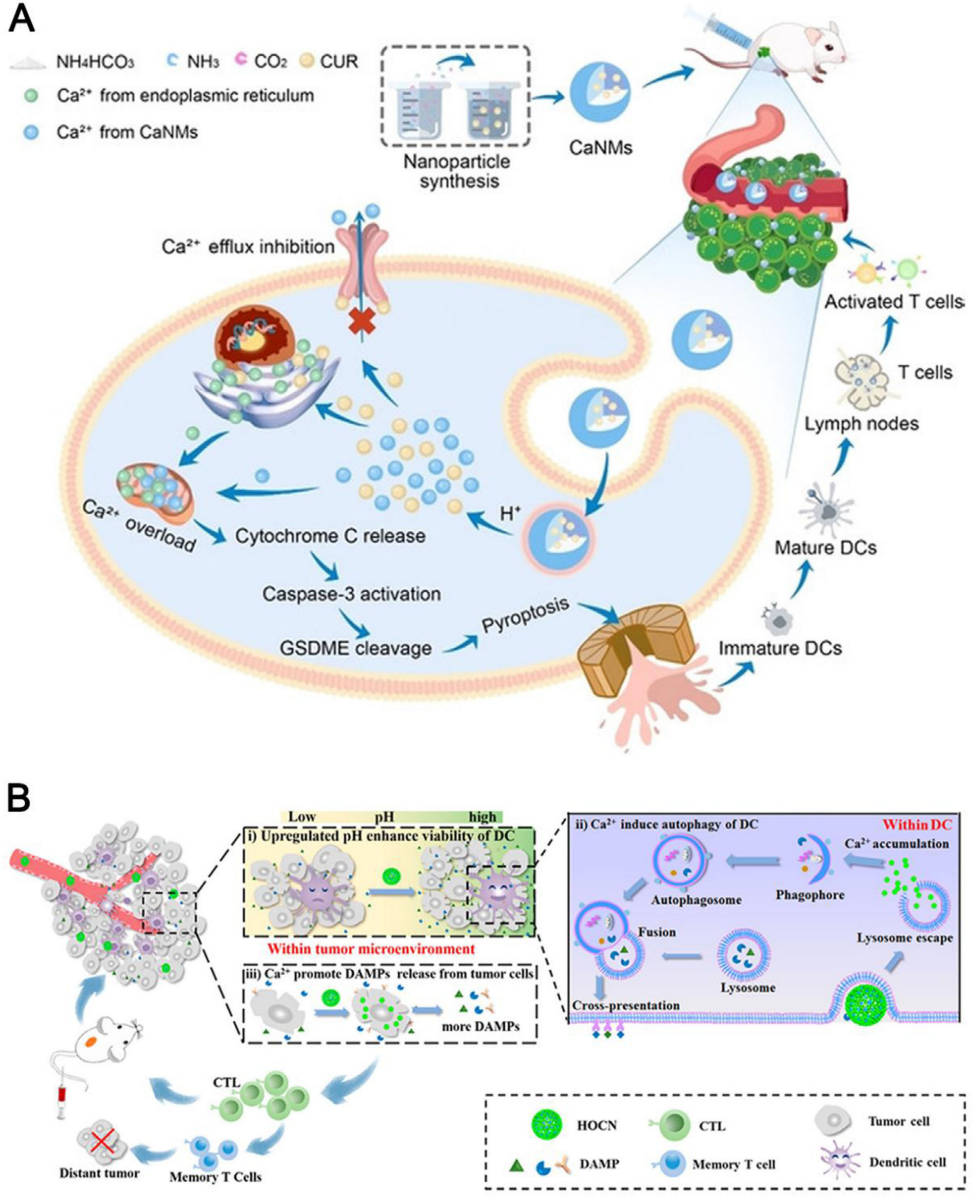
(A) Schematic illustration of Ca^2+^ nanomodulator‐induced pyroptosis for cancer immunotherapy. Reprinted with permission.^[^ ^12c^ ^]^ Copyright 2022, Wiley. (B) Schematic illustration of OVA@CaCO_3_ enhanced APC maturation for chemoimmunotherapy. Reprinted with permission.^[^ ^12a^ ^]^ Copyright 2020, Elsevier.

Ca^2+^ has also been proven to increase intracellular ROS levels by causing mitochondria dysfunction and ER stress, resulting in ICD. The anti‐tumor immune responses elicited by ICD are unstable since DAMPs released from dying cells are sometimes insufficient to elicit adequate immune stimulation for DC maturation.^[^ ^76^ ^]^ Therefore, increasing the amounts of DAMPs released from dying cells is expected to promote the antigen presentation performance of DCs. Various studies reported that intracellular calcium overload leads to DAMPs exposure by causing mitochondrial injury and oxidative ER stress, leading to amplified ICD.^[^ ^12^, ^76^, ^77^ ^]^ Besides the induction of typical ICD and apoptosis, calcium overload was also reported to elicit GSDME‐dependent pyroptosis in a recent study (Figure 9A).^[^ ^12c^ ^]^ Mechanistically, the introduction of abundant Ca^2+^ into mitochondria resulted in an imbalance of Ca^2+^ homeostasis and mitochondrial Ca^2+^ overload, leading to the release of cytochrome C which enables the activation of caspase‐3 and subsequent cleavage of GSDME. In this study, they demonstrated that Ca^2+^ overload induced pyroptosis via cytochrome C‐caspase‐3‐GSDME pathway. Finally, the cleaved GSDME perforated the cellular lipid membrane, causing an outflow of abundant intracellular proinflammatory molecules and dying cellular components, which intensively induced APC maturation and T‐cell activation. As a result, the innate and adaptive immune systems were activated to destroy cancer cells.

In addition to induce ICD, intracellular Ca level was also demonstrated to be positively correlated with DC autophagy process which aids in DC maturation by storing antigens in the cytoplasm for reprocessing.^[^ ^78^ ^]^ An and co‐workers validated that honeycomb OVA@CaCO_3_ nanoparticles (NPs) promoted the DC autophagy level as revealed by enhanced co‐localization of antigen and autophagosome (up to 84%) and increased expression of MHC‐II and CD86 (up to 48.87%), compared to the other groups (Figure 9B).^[^ ^12a^ ^]^ In summary, intracellular Ca^2+^ overload can lead to antitumor immunity via multiple ways, including direct triggering and amplifying typical ICD, activating cytochrome C‐caspase‐3‐GSDME‐pyroptosis pathway, and promoting DC autophagy for DC maturation and antigen presentation.

#### 2.4.2 Ca‐containing nanomaterials

Regarding Ca‐containing nanomaterials, CaCO_3_ NPs are the most used materials in nanomedicine and Ca‐induced metalloimmunotherapy. Zheng et al. devised an all‐immunity‐phase‐boosted strategy by encapsulating Mn ion‐doped CaCO_3_ NPs and CAIX inhibitor SLC‐0111 (SLC) into palmitoyl ascorbate (PA)‐liposome (PL) (Mn/CaCO_3_@PL/SLC).^[^ ^79^ ^]^ Mn^2+^ on the one hand in tandem with Ca^2+^ promoted ROS production to induce tumor ICD for initiating the CIC, on the other hand directly stimulated the Cgas/STING signaling pathway to boost the all‐immunity‐cycle. Additionally, SLC as a TME modifier and PA as an immune enhancer collaboratively aided in the antitumor immune responses. In another study, CaCO_3_ NPs were used to load a Ca^2+^ enhancer (curcumin (CUR)) and coated with a Ph‐sensitive PEG shell.^[^ ^12b^ ^]^ This study demonstrated that the Ph‐responsive release of Ca^2+^ from CaCO_3_ NPs synergized with CUR and ultrasound, leading to mitochondrial Ca^2+^ overload and efficient tumor cell apoptosis. Specifically, they found that Ca^2+^ overload led to tumor ICD as revealed by increasing expression levels of CRT, HMGB1, and CRT. In a follow‐up study, they reported that in addition to ICD effect, CaCO_3_ NPs in combination with CUR also caused GSDMD‐dependent pyroptosis which also caused antitumor immunity demonstrated by a significantly higher level of DC maturation and CTL frequency compared to the control group.^[^ ^12c^ ^]^

### 2.5 Zinc (Zn)

Zn is referred to as a trace element, as its plasma concentration is only 10–20 mm. Zn^2+^ serves as the catalytic core or structural stabilizer in over 300 enzymes and is a component of an even larger number of additional metalloproteins. The immune system is regulated delicately by zinc homeostasis. Similar to Ca^2+^, Zn^2+^ functions as a second messenger in innate immunity. In the adaptive immune system, pre‐T cells are most susceptible to zinc deficiency. In this part, we summarized the major signaling pathways involving Zn^2+^ in antitumor immune responses and introduced Zn‐containing nanomaterials that can assist in delivering Zn element into the tumor site precisely without inducing systemic free metal toxicity.

#### 2.5.1 Immune‐related pathways

##### ICD pathways

Similar to Ca^2+^, disrupting intracellular Zn^2+^ homeostasis has been documented to increase intracellular ROS levels by causing mitochondria dysfunction and ER stress, resulting in ICD induction for evoking systematic antitumor immunity.^[^ ^80^ ^]^

##### STING pathways

Nutritional Zn^2+^ was also reported to activate the cGAS‐STING signaling pathway in tumor cells.^[^ ^11b^ ^]^ However, the activation mechanism differs from Mn^2+^‐mediated cGAS‐STING activation, which only relies on the STING upstream. At the start of the cGAS‐STING signaling pathway cGAS detects cytosol dsDNA and forms dsDNA/cGAS oligomeric complexes which undergo liquid−liquid phase separations within the cytosol, forming liquid‐like droplets that function as intracellular microreactors for 2′3′‐cGAMP production. Du et al. reported that Zn^2+^ at ∼200 mm dramatically facilitated DNA‐induced cGAS phase separation at low concentrations of cGAS and DNA and then catalyzed cGAMP synthesis rapidly. The Zn^2+^‐mediated cGAS‐STING activation for generating antitumor immunogenicity was corroborated by a recent study in which Zn^2+^ synergized with ROS‐induced cytosol DNA exposure to activate the cGAS‐STING pathway for combating liver cancer through nanocomposites.

#### 2.5.2 Zn‐containing nanomaterials

**FIGURE 10:**
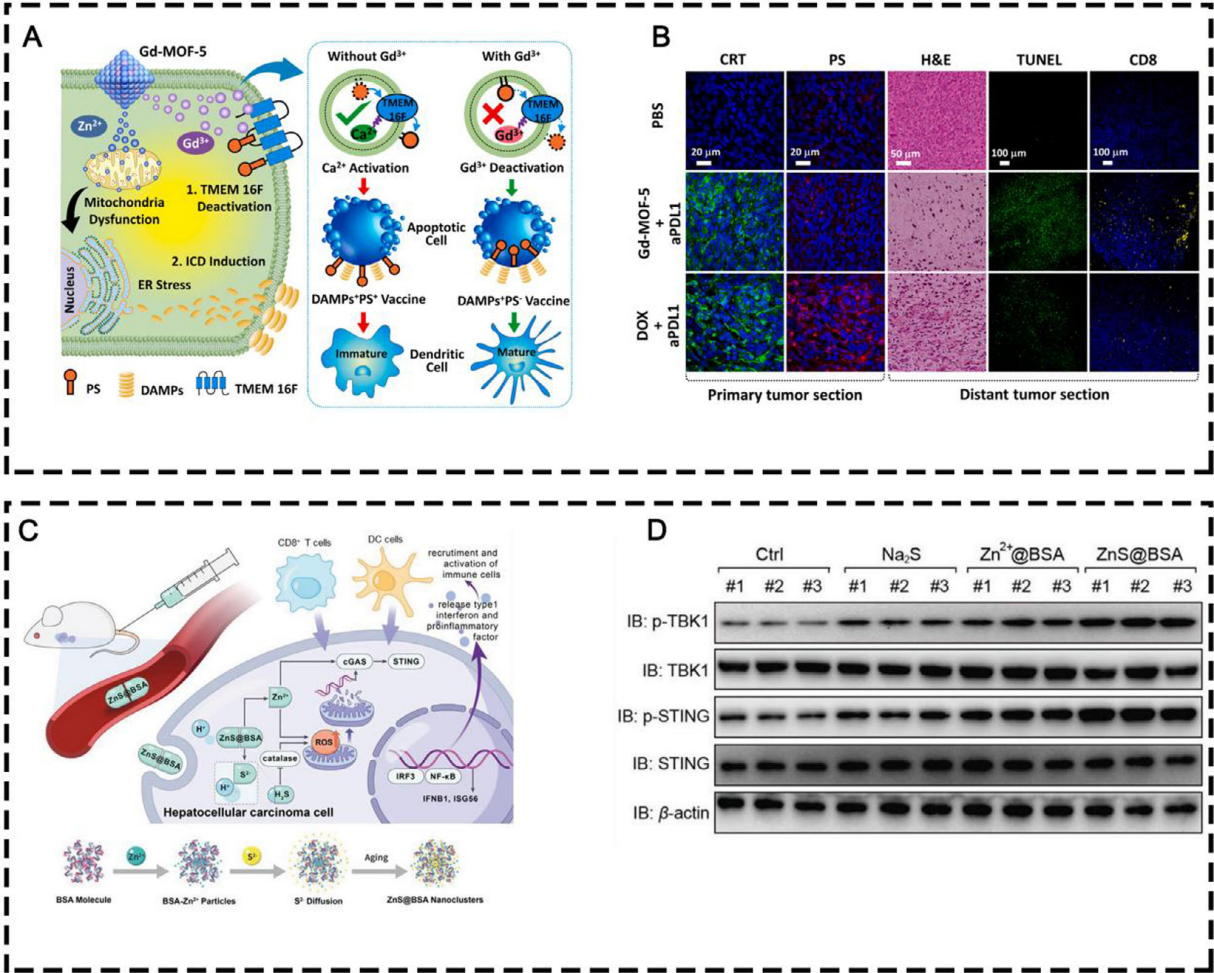
(A) Schematic illustration of Gd‐MOF‐5 NPs in modulating immunosuppressive PS and ICD signals. (B) CRT and PS staining of primary tumors and H&E, TUNEL, and CD8 staining of distant tumors with different treatments. Reprinted with permission.^[^ ^82^ ^]^ Copyright 2021, Elsevier. (C) The therapeutic illustration of ZnS@BSA nanoclusters and the fabrication of ZnS@BSA nanoclusters. (D) Western blot analysis of protein levels in cGAS‐STING pathway in randomly selected tumor samples after different treatments. Reprinted with permission.^[^ ^84^ ^]^ Copyright 2021, Wiley.

Zinc oxide NPs have been widely explored to induce ROS‐induced ICD through catalyzing Fenton‐like reactions and disrupting mitochondria function.^[^ ^81^ ^]^ Regarding organic‐inorganic hybrid nanomaterials, Dai and co‐workers reported bimetallic nMOF NPs containing both Gd^3+^ and Zn^2+^ (Gd‐MOF‐5) that could be used as immunomodulators to potentiate the therapeutic efficacy of anti‐PD‐L1 antibodies (Figure 10A,B).^[^ ^82^ ^]^ Mechanistically, Gd^3+^ down‐regulated the immunosuppressive PS signal and Zn^2+^ induced the intracellular ROS production and ICD to up‐regulate immunostimulatory signals. The Gd‐MOF‐5 nanostructures effectively inhibited both primary and distal tumor growth in bilateral 4T1 tumor‐bearing mouse models. In addition, an interesting paper reported that Zn^2+^ could be doped into layered double hydroxide (LDH) through a Zn‐LDH bond and be applied peritumorally to activate cGAS‐STING signaling pathway, thus promoting antitumor immunity.^[83]^

In addition, Cen *et al.* developed ZnS@BSA nanoclusters in which BSA co‐loaded zinc and sulfur via an ion diffusion approach for liver cancer immunotherapy (Figure 10C,D).^[^ ^84^ ^]^ Sulfur ions and intracellular hydrogen ions were the sources of intracellular H_2_S formation, leading to ROS production which was also potentiated by Zn^2+^. The cytosol DNA damage and exposure induced by ROS and Zn^2+^ synergistically activated the cGAS‐STING pathway for antitumor immunogenicity, leading to effective suppression of the primary and distant Hepa1‐6 tumor growth.

### 2.6 Sodium (Na)

Na^+^ participates in a lot of biological activities in all living organisms, including osmotic equilibrium and acid‐base balance. Perturbation of cellular and systemic osmotic pressure equilibrium severely challenges the function of all organisms.^[^ ^85^ ^]^ Under physiological conditions, it is difficult to elevate intracellular osmolarity, since cells maintain ion homeostasis which is strictly regulated by ion pumps and channels that are anchored on cell membranes. However, recent studies proposed that metal ions could cause intracellular osmolarity surge in tumor cells via nanotechnology, and then give rise to an unusual manner of cell death: immunogenic caspase‐1‐related pyroptosis.^[^ ^13^, ^86^ ^]^ In this section, we will introduce Na^+^‐induced immune stimulative ways to inhibit tumor growth and nano‐related materials that can help deliver Na^+^ into the TME.

#### 2.6.1 Immune‐related pathways

##### ICD pathways

Pyroptosis, a pro‐inflammatory form of ICD, is characterized by swollen and lysed cell structure, pro‐inflammatory cytokines (IL‐1β, IL‐18) release, ATP generation, and HMGB1 expression that initiate the innate and adaptive immune systems, leading to systemic antitumor immunogenicity.^[^ ^87^ ^]^

**FIGURE 11:**
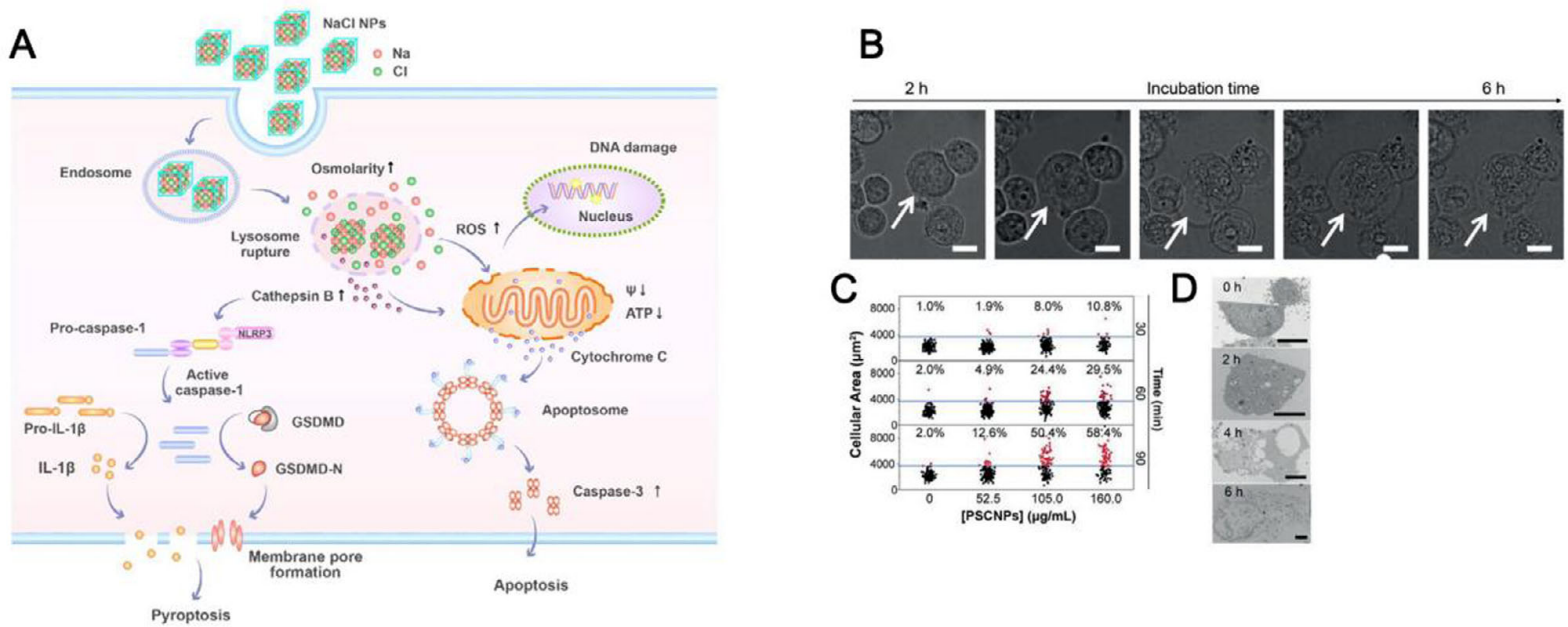
(A) Schematic illustration of the mechanism of NaCl NP‐induced pyroptosis. (B) Cell morphology changes overtime with PSCNPs. Scale bar, 50 µm. (C) Cell volume changes based on the statistics of 5000 cells. (D) Representative TEM images of PSCNP‐treated PC‐3 cells. Scale bar, 5 µm. Reprinted with permission.^[^ ^13^ ^]^ Copyright 2018, Wiley.

Jiang et al. reported that the introduction of excessive Na^+^ into tumor cells disrupted intracellular osmolarity homeostasis, and further activated caspase‐1‐related pyroptosis, leading to the release of the N‐terminal of gasdermin‐D (GSDMD) proteins, which can translocate to the plasma membrane and perforate it, causing cell rupture and outflow of immunogenic mediators (Figure 11).^[^ ^13^ ^]^ Moreover, they found much better treatment outcomes in immunocompetent mice than in immunodeficient mice. They discovered increased surface presentation of CRT, elevated secretion of ATP, and HMGB‐1 in tumor tissue after Na^+^ treatment, thus potentially amplifying the therapeutic efficacy of ICBs, such as anti‐CTLA‐4 or anti‐PD‐1/PD‐L1 antibodies. This specific mechanism of Na^+^‐induced immunogenic pyroptosis was also corroborated by two follow‐up studies.^[^ ^86^ ^]^

#### 2.6.2 Na‐containing nanomaterials

##### Inorganic nanomaterials

Electrolyte NPs have been underestimated in the field of nanomedicines because electrolyte NPs are unstable and easy to degrade since they would quickly dissolve in water and behave similarly as their constituent salts, thereby shortening in vivo circulation time. Na‐based electrolyte NPs have garnered increasing interest in cancer therapy in recent years due to their promising anti‐tumor effects.

**FIGURE 12:**
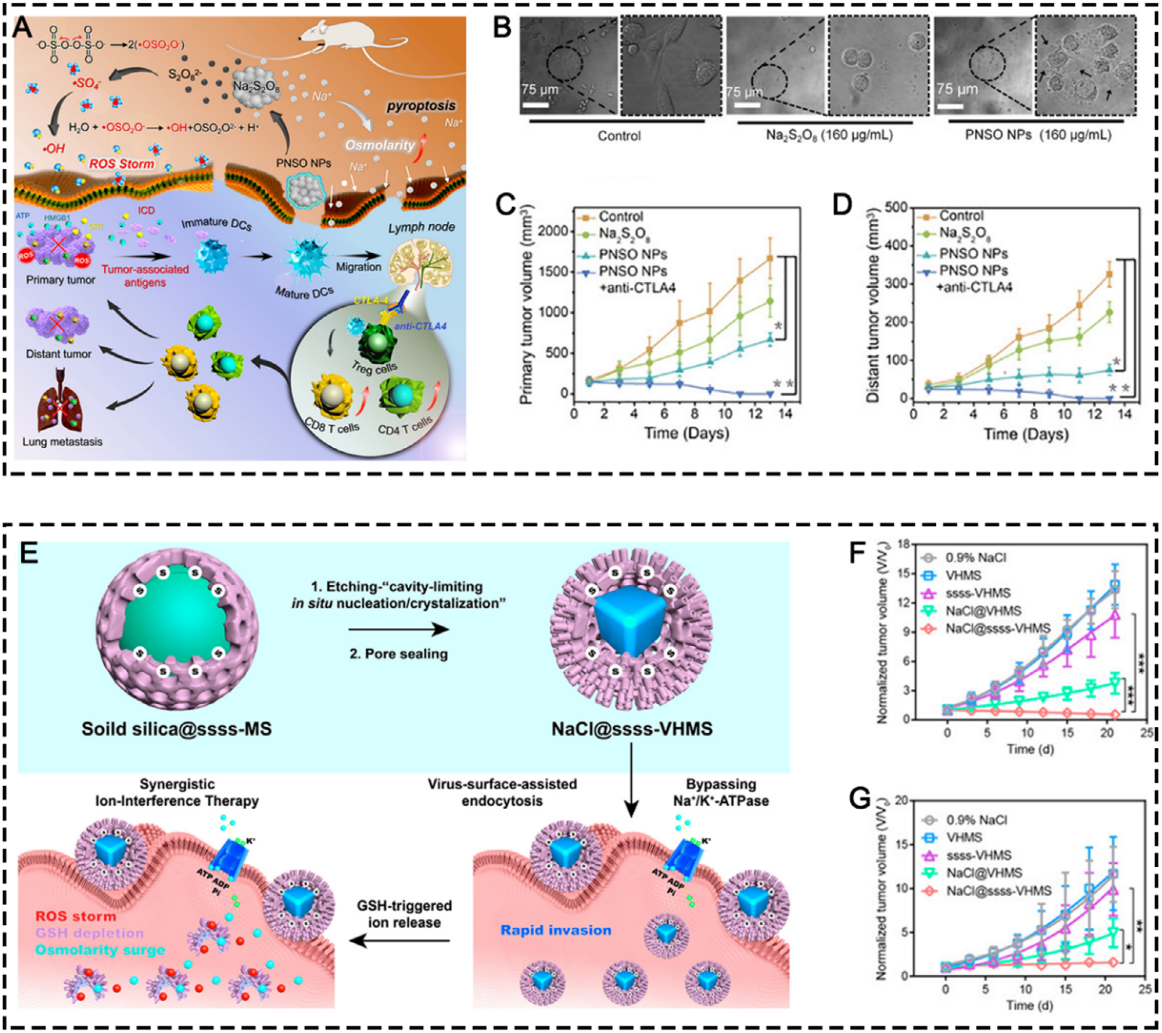
(A) The schematic illustration of the therapeutic process mediated by PNSO NPs. (B) Morphology of 4T1 cells after different treatments. The antitumor growth curve of (C) the primary and (D) the distant tumors in different treatment groups, including the control, Na_2_S_2_O_8_, PNSO NPs, and PNSO NPs+anti‐CTLA4 groups. Reprinted with permission.^[^ ^86a^ ^]^ Copyright 2020, American Chemical Society. (E) The synthetic process of NaCl@ssss‐VHMS and its illustrative therapeutic mechanism. The antitumor growth curve of (F) HepG2 tumor‐bearing mice and (G) 4T1 tumor‐bearing mice in different treatment groups, including 0.9% NaCl, VHMS, ssss‐VHMS, NaCl@VHMS, and NaCl@ssss‐VHMS groups. Reprinted with permission.^[^ ^86b^ ^]^ Copyright 2022, American Chemical Society.

Sodium chloride NPs (SCNPs) were exploited as a Trojan horse to carry abundant free Na^+^ into various cells and thus disrupt intracellular ion homeostasis. In 2019, Jiang et al. synthesized SCNPs through a microemulsion reaction and coated a layer of PEGylated phospholipid, DSPE‐PEG2000 amine, onto the NP surface for extending the NP lifetime (Figure 11).^[^ ^13^ ^]^ The synthesized SCNPs were demonstrated to disrupt the intracellular ion homeostasis and cause a surge of osmolarity and rapid cell lysis, leading to caspase‐1‐related pyroptosis which is a type of ICD. The SCNPs were demonstrated to elicit tumor regression via intratumoral injection in various tumor models, which were highly immunogenic and stimulated significant anti‐tumor immune responses. In a follow‐up study, phospholipid‐coated Na_2_S_2_O_8_ NPs (PNSO NPs) were developed to deliver large amount of Na^+^ into tumor cells for triggering caspase‐1‐related pyroptosis (Figure 12A–D).^[^ ^86a^ ^]^ In addition, the PNSO NPs were able to release S_2_O_8_^2−^ which was further changed into toxic •SO^4−^ (a novel reported ROS) and •OH for ROS‐induced ICD which worked jointly with pyroptosis to maximize the anti‐tumor immunogenicity, leading to efficient suppression of the primary and distant tumors. However, either the phospholipid coated SCNPs or PNSO NPs were prone to degrade in water within a few hours even if they were protected by phospholipid shells. To address this issue, Yang and co‐workers synthesized NaCl nanocrystals that were wrapped by GSH‐responsive virus‐inspired hollow mesoporous tetrasulfide‐organosilica (ssss‐VHMS) and further protected by phospholipid and bifunctional PEG (silane‐PEG‐silane) to enclose the pore and thus prevent the premature leakage and release of NaCl nanocrystals (Figure 12E–G).^[^ ^86b^ ^]^ This TME‐responsive NaCl@ssss‐VHMS nanostructure was demonstrated to elicit apoptosis, ferroptosis, and pyroptosis, which concurrently resulted in significant tumor inhibition in both 4T1 and HepG2 tumor‐bearing mice via intravenous injection.

### 2.7 Gadolinium (Gd)

Gadolinium (Gd^3+^) is a lanthanide heavy metal ion with high charge density. Ga‐containing compounds are classical MRI contrast agents in clinical settings. Other than that, Ga element was found to relate to the innate and immune systems and may contribute to antitumor immunity.

As we mentioned earlier, the ICD effect of tumor cells can be influenced by externalization of phosphatidylserine (PS) on the cancer cell surface, which can interact with receptors on numerous immunosuppressive cells, leading to the release of immunosuppressive cytokines (TGF‐β and IL‐10). In addition, PS‐expressing cells hinder DC maturation, thereby preventing antigen presentation to naïve T cells.^[^ ^88^ ^]^ Normally, the PS remains inside the plasma membrane. In the apoptotic state, high levels of Ca^2+^ ions accumulate in the cytosol and activate PS scramblase to transfer PS to the outer side of the cell membrane.^[^ ^82^ ^]^ The stronger binding affinity of Gd^3+^ over Ca^2+^ inhibits the biological processes requiring Ca^2+^ or the functions of Ca^2+^ binding enzymes.^[^ ^89^ ^]^ Therefore, a preclinical study demonstrated that a Gd‐based metal‐organic framework was able to reduce the enzymatic activity of PS scramblase through competitive binding and inhibiting PS externalization, thereby amplifying the antitumor immunity caused by ICD (Figure 10A,B).^[^ ^82^ ^]^ Although these studies indicated Gd‐based composites possess potential anti‐tumor immune‐activating properties, the corresponding molecular mechanisms entail in‐depth exploration. Besides, a lot of studies are required to be done to determine the antitumor immunogenicity of Gd‐based materials in various types of tumors.

### 2.8 Platinum (Pt)

In recent years, Pt‐based metal compounds, including diaminocyclohexane‐Pt (II), and DACH‐Pt (II), were reported to boost antitumor immunity via numerous ways. Besides, Pt^2+^ present in Pt‐based nanomaterials can increase intracellular ROS levels by causing mitochondria dysfunction and ER stress, resulting in ICD induction and enhanced antitumor immunity. Hence, we made a short summary about Pt‐based metallodrug and Pt‐based nanomaterials in inducing antitumor immunity.

For example, Pt^4+^ was coordinated with camptothecin (CPT), followed by self‐assembly with a ROS‐sensitive copolymer and a lipid polymer (mPEG2k‐DSPE) to develop a ROS‐responsive nanodrug, denoted as CPT‐Pt (IV).^[^ ^90^ ^]^ The Pt‐based hybrid nanodrug was proven to activate the forefront of the cGAS‐STING signaling pathway by ROS‐induced DNA damage and dsDNA cytosol exposure first, leading to both short and long‐term anti‐tumor immune responses and effective suppression of CT26 tumor growth. Besides, diaminocyclohexane‐Pt (II) was used to coordinate with TA with the help of Fe^3+^ to reinforce the structure of the TA‐Pt framework and enhance the colloidal stability of the nanoformulations. The TA‐Pt NPs were proved to induce ICD and reverse the immunosuppressive TME, leading to effective CT26 tumor regression. Hueng et al. coordinated DACH‐Pt (II) with mPEG‐PKS to develop nanocomposites with phago‐/endosome escape by introducing thioether groups and lysine molecules.^[^ ^91^ ^]^ In response to intratumoral excessive H_2_O_2_, Pt‐based drugs were released into the TME from the nanocomposites and caused significant ROS production in both tumor cells and TAMs, leading to tumor cell ICD and TAM polarization, respectively, and ultimately resulting in tumor regression. In addition, Pt NPs were demonstrated to possess CAT activities and led to relieved intratumoral hypoxia and TAM polarization.

### 2.9 Potassium (K)

In a study that explored the biological effect of gasdermin D, Banerjee and coworkers found that similar to Mn^2+^ and Zn^2+^ that can increase the enzymatic activity of cGAS and its sensitivity for cytosol DNA, K^+^ can also facilitate cGAS‐DNA interaction and enhance its enzymatic function, and therefore increase the production of type I IFN.^[^ ^92^ ^]^ Mechanistically, they found that gasdermin D activated by the AIM2 inflammasome caused abundant K^+^ efflux through forming pores in the cell membrane, while blockade of K^+^ efflux enhanced type I IFN. More specifically, they demonstrated that gasdermin D‐induced K^+^ efflux targeted cGAS to inhibit cytosol DNA‐led type I IFN production by decreasing cGAS‐DNA affinity, cGAS oligomerization, and enzymatic activity of cGAS. Given the great potential of STING activation for assisting cancer immunotherapy, it is worth exploring how K^+^ can contribute to anti‐tumor immune responses.

Aside from its regulatory role on the cGAS‐STING pathway, a high concentration of intratumoral K^+^ was demonstrated to maintain T‐cell stemness by restricting T‐cell nutrient intake, thereby inducing autophagy and reducing histone acetylation at effector and exhaustion loci.^[^ ^93^ ^]^ The author named the restricted nutrient intake caused by enhanced intratumoral K^+^ under abundant extracellular nutrient supply as caloric restriction. Although T cells with stem cell‐like behavior are not able to exert effector function with respect to cytokine production and cytotoxicity, they have self‐renewal and multipotent performance and may help eradicate large metastatic tumors in combination with mainstream immunotherapy, such as ICB. The immuno‐regulatory role played by K^+^ provides new insights for next‐generation immunotherapy with an eye on maintaining T‐cell stemness. However, another study reported that a high concentration of intratumoral K^+^ suppressed the antitumor effect of TAMs and inhibited the polarization of TAMs into M1 phenotype via the inwardly rectifying K^+^ channel Kir2.1 which can sense high K^+^ in the TME and control TAM metabolic condition between glutaminolysis and glycolysis, and thereby affect TAM functions.^[^ ^94^ ^]^ Hence, considering both the protumoral role and antitumoral role played by K^+^, cautions are needed when manipulating K^+^ for antitumor purposes. Furthermore, one needs to identify which signaling pathway plays the major role in K^+^‐induced anti‐tumor process.

### 2.10 Rhodium (Rh)

Rh‐related metallodrugs have shown great promise in anticancer activities by interfering with oncogenic signaling and cancer‐promoting regulatory processes.^[^ ^95^ ^]^ Regarding immune regulation, although some Rh complexes play anti‐inflammatory roles by inhibiting the activity of various immune cells (macrophages) and down‐regulating cytokine production, other Rh compounds were reported to possess immune‐stimulatory effects by activating dentritic cells and T cells.^[^ ^96^ ^]^ To summarize, immune‐inhibitory or ‐stimulatory effects are the result of the pleiotropic effects of Rh compounds on different immunocyte, which are also dependent on specific pathophysiological conditions. With respect to immunogenic effect of Rh‐related complexes or nanomaterials for anticancer immunotherapy, there are literally no relevant reports and rare exploration. As reviewed by Walter Berger, considering the prominent anti‐inflammatory role played by Rh complexes and low catalytic activity for ROS production, the immunogenic activity of Rh‐containing complexes or nanomaterials is greatly limited.^[^ ^8^ ^]^

### 2.11 Arsenic (As)

In contrast to most other metals discussed in this review, As is a metalloid that has properties intermediate between metals and nonmetals. In specific chemical environment, As can display some metal characteristics and can form metallic compounds and be alloyed with other metals. Hence, in some circumstances, scientists categorize As compounds as metal‐based drugs. The most commonly used clinical As‐containing drug is arsenic trioxide (As_2_O_3_, ATO) which has been widely used for treating acute promyelocytic leukemia (APL) and explored for other cancer types.^[^ ^97^ ^]^ With regard to immune compartment, As and As compound were documented to exert immunosuppressive effect in autoimmune reactions. However, in immune‐disregulated TME, As compounds were reported to stimulate the immune landscape to inhibit tumor growth via depleting Treg cells, reducing MDSCs, enhancing NK cell activities, and stimulating CTL response. The specific mechanisms were summarized in a previous review.^[^ ^8^ ^]^ With regarding to As‐related nanomedicine, nanomaterials, such as liposome, were applied to deliver ATO to overcome its high systemic toxicity, rapid renal elimination, and low tumor selectivity. Excepting wrapping ATO into nanocarriers, nanomaterials that were composed of pristine monoelemental arsenic were reported to feature outstanding antitumor activity and immune activation.^[^ ^98^ ^]^ Recently, Guo and coworkers fabricated 2D arsenene nanosheets and explored the corresponding anticancer mechanism and immune regulatory capability.^[^ ^99^ ^]^ By single‐cell RNA sequencing and proteomic analysis, they revealed that arsenene nanosheets specifically inhibited thioredoxin TXNL1 to disrupt the intracellular redox balance, leading to the overloaded oxidative stress and cellular ROS which further induced immunogenic cell death, DC maturation, and immune cell recruitment.

### 2.12 Molybdenum (Mo)

Mo is a transition metal and an essential trace element for humans. Knowledge about the association of Mo and immune activities is rather vague and fragmentary. In anticancer treatment, Mo is mainly applied in the form of tetrathiomolybdate, which was validated to antagonize inflammation and angiogenesis.^[^ ^8^ ^]^ With regard to effects of Mo compounds on anticancer immunity, this field is literally unexplored. However, with an eye on Mo‐based nanomedicine, Mo‐containing inorganic nanomaterials were frequently used for chemodynamic therapy due to Fenton like reaction since Mo always cycles between the oxidation state +4 and +6.^[^ ^100^ ^]^ As demonstrated by Lin and coworkers, the synthesized Cu_2_MoS_4_ realized strong CDT and elicited vaccine‐like antitumor immune responses after primary tumor ablation.^[^ ^72^ ^]^ As summarized by Akhilesh K. Gaharwar and co‐workers, MoS_2_‐based ultrathin nanomaterials were proved to modulate multiple immune compartments in the TME, including polarizing macrophages into M1 phenotype, promoting DC maturation.^[^ ^101^ ^]^ Specifically, absorbing protein corona and passivating MoS_2_ with ligands such as PEG displayed stronger immune activation than pristine MoS_2_. With regard to molecular mechanism, MoS_2_ mediated intracellular ROS generation was believed to be responsible for macrophage polarization and DC maturation.

## 3 THERANOSTIC POTENTIALS OF SMART METAL NANOSYSTEMS

Some metals such as Fe, Mn, Cu, and Gd endow MSNs the imaging performance properties, therefore, MSNs have been enthusiastically explored for immunotheranostic purposes. In this section, we mainly summarized the theranostic applications of some popular MSNs.

### 3.1 Fe‐based MSNs as immunotheranostics

**FIGURE 13:**
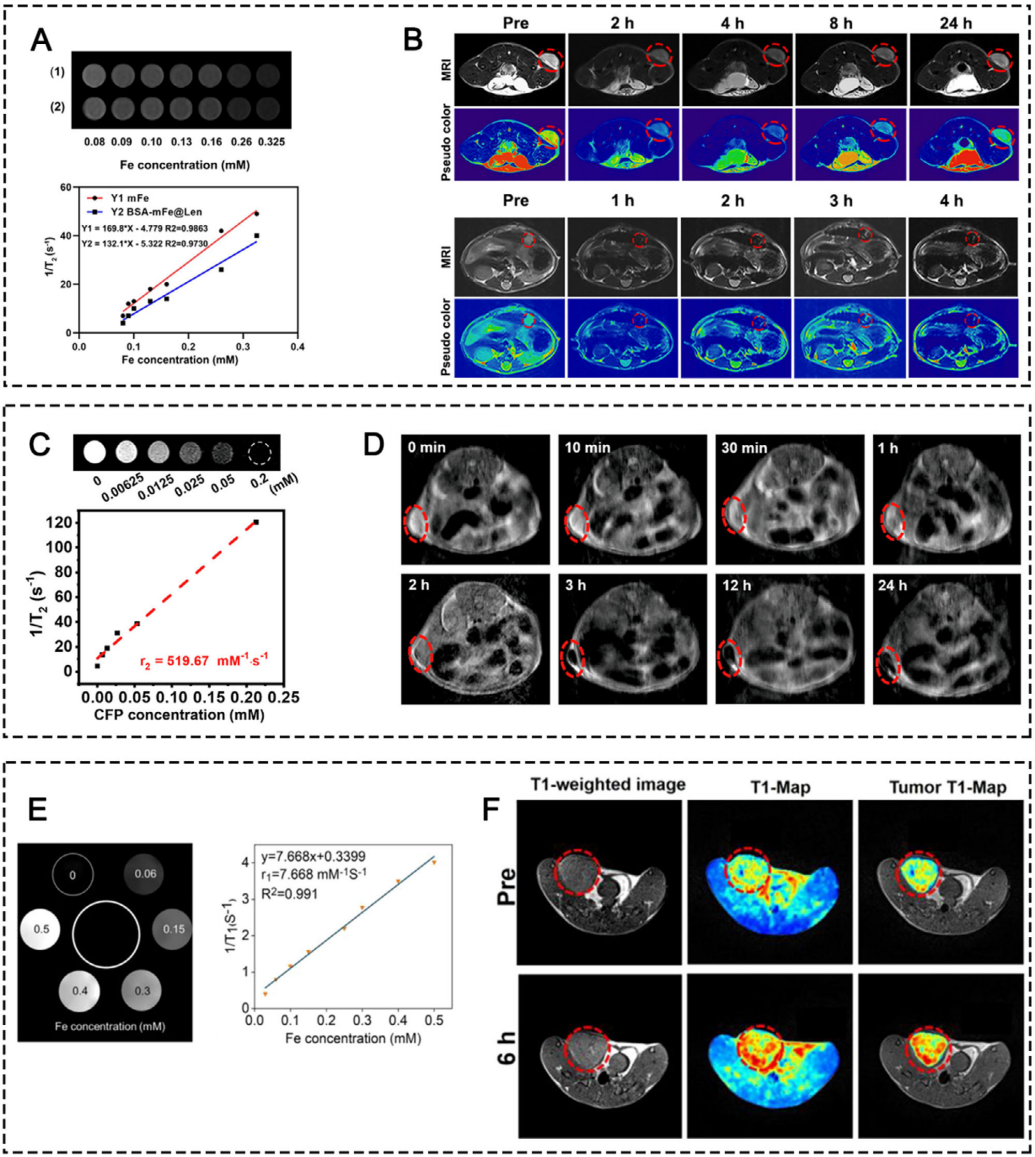
(A) *T*_2_‐weighted MRI phantom images and the counted r_2_ values of (1) the mFe and (2) BSA‐mFe@Len NPs with gradient Fe concentrations. (B) *T*_2_ weighted grayscale MRI and pseudo color images of the subcutaneous and orthotopic tumor models after intravenous injection of BSA‐mFe@Len NPs. Reprinted with permission.^[^ ^44c^ ^]^ Copyright 2022, Elsevier. (C) *T*_2_‐weighted MRI phantom images and the counted *r*_2_ value of the CFP within gradient Fe concentrations. (D) *T*_2_ weighted grayscale MRI of tumor regions at different time points after intravenous injection of CFP. Reprinted with permission.^[^ ^47^ ^]^ Copyright 2022, American Chemical Society. (E) *T*_1_‐weighted MRI phantom images and the counted *r*_1_ value of the Fe‐PDA within gradient Fe concentrations. (F) *T*_1_‐weighted MRI of tumor regions at different time points after intravenous injection of Fe‐PDA‐mPEG. Reprinted with permission.^[^ ^44d^ ^]^ Copyright 2022, American Chemical Society.

Due to superparamagnetic behavior, Fe_3_O_4_ NPs can be employed as MRI contrast agents by shortening the relaxation time of surrounding protons. Fe_3_O_4_ NPs mainly show a shortening effect on *T*_2_/*T*_2_* relaxation which leads to a decreased signal intensity on *T*_2_/*T*_2_*‐weighted images. Thus Fe_3_O_4_ NPs are commonly used as MRI *T*_2_‐weighted imaging agents and their dark contrast imaging performance has been evaluated in numerous preclinical studies.^[^ ^45^ ^]^ In some studies that explored the Fe_3_O_4_‐induced antitumor immunity, the *T*_2_‐weighted negative MRI performance was also successfully demonstrated in vitro and in vivo, indicating the versatility of Fe_3_O_4_ NPs as cancer immunotheranostics.^[^ ^24^, ^41^, ^44^, ^46^, ^102^ ^]^ Our research team has been devoted to exploring the negative contrast performance of Fe_3_O_4_ NPs in cardiovascular diseases and cancers for decades.^[^ ^103^ ^]^ Very recently, we showed that the as‐prepared BSA‐mFe@Len NPs not only generated great tumor inhibition effects by the concerted efforts of Fe_3_O_4_ NPs‐induced metalloimmunotherapy and Len‐induced tumor vessel normalization, but also exerted prominent *T*_2_‐weighted MRI performance. The BSA‐mFe@Len NPs were able to darken the tumor site in both subcutaneous and orthotopic liver cancer models, with the *r*_2_ value estimated to be 132.1 mm/s, suggesting the immunotheranostic potential of the BSA‐mFe@Len NPs (Figure 13A,B).^[^ ^44c^ ^]^

Iron oxide analogues have also been investigated as MR imaging contrasts owing to their favorable superparamagnetic properties. For example, CoFe_2_O_4_ is an iron oxide analogue and also a potent catalyst for ROS production because of its multivalent elements (Co^2+^/Co^3+^, Fe^2+^/Fe^3+^)^[^ ^104^ ^]^. The PEGylated CoFe_2_O_4_ nanoflowers (CFPs) reported by Fu et al. induced SDT and CDT for boosting antitumor immunity.^[^ ^47^ ^]^ Furthermore, they found that the CFPs were able to shorten the spin−spin relaxation time (*T*_2_) and displayed significant negative MRI performance with *T*_2_ relaxivity (*r*_2_) calculated to be 519.67 mm/s, indicating that CFPs are potential MRI imaging contrast agents for clinical diagnosis (Figure 13C,D).

In addition to the superparamagnetism of iron oxide or iron oxide analogues, the existence of Fe^3+^ itself possesses a *T*_1_‐weighted MRI contrast‐enhancement performance.^[^ ^105^ ^]^ As an example, Xu et al. reported a Fe^3+^‐chelated PDA NP for cancer immunotherapy and photothermal therapy.^[^ ^44d^ ^]^ The Fe^3+^‐chelated PDA NPs were demonstrated to possess *T*_1_‐weighted MRI performance in vitro and in vivo, with *r*_1_ measured to be 7.668 mm/s, indicating that the Fe^3+^‐chelated PDA NPs could efficiently aggregate in the tumor and act as an excellent *T*_1_‐MRI contrast for tumor diagnosis (Figure 13E,F).

### 3.2 Mn‐based MSNs as immunotheranostics

Mn‐based MSNs have been widely applied in *T*_1_‐weighted MRI monitoring to achieve theranostic purposes.^[^ ^106^ ^]^ Manganese oxides (MnO_2_, MnO, MnO_x_), manganese phosphate, manganese coordination copolymer, and manganese ion loaded/doped nanomaterials all possess *T*_1_‐weighted MRI performance for tumor diagnosis in a TME‐responsive manner due to the presence of Mn^2+^.^[^ ^24^, ^25^ ^,g,^ ^40^, ^107^ ^]^

**FIGURE 14:**
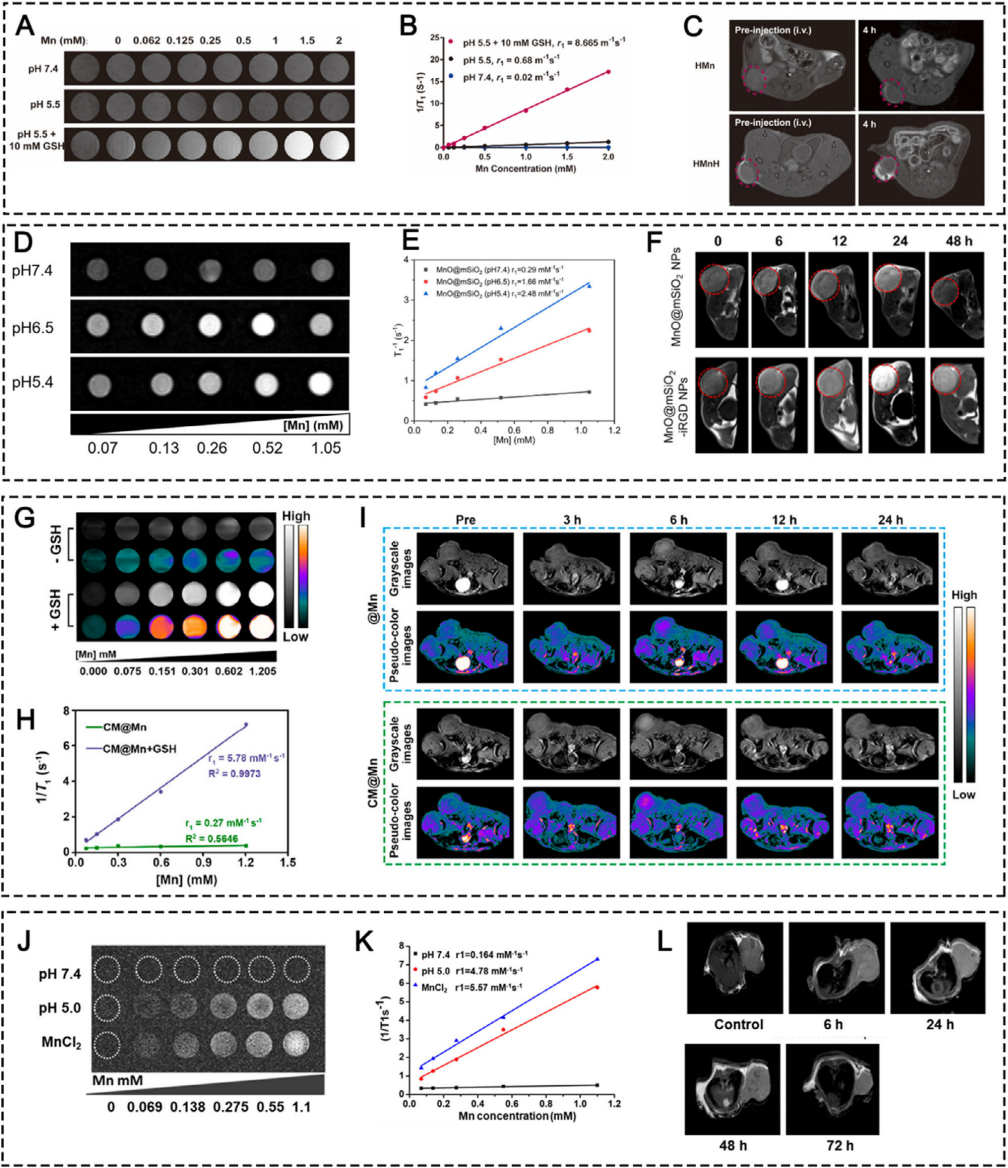
(A) *T*_1_‐weighted grayscale MRI of the HMn NPs with different Mn concentrations in the solution with pH 7.4, pH 5.5, pH 5.5 + 10 mm GSH and (B) the corresponding *r*_1_ values. (C) In vivo *T*_1_‐weighted MRI before and after intravenous injection of HMn or HMnH NPs. Reprinted with permission.^[^ ^107c^ ^]^ Copyright 2022, Elsevier. (D) *T*_1_‐weighted grayscale MRI of the MnO@mSiO_2_ NPs with different Mn concentrations in the solution with pH 7.4, pH 6.5, pH 5.4, and (E) the corresponding *r*_1_ values. (F) In vivo *T*_1_‐weighted MRI before and after intravenous injection of MnO@mSiO_2_ NPs or MnO@mSiO_2_‐iRGD NPs at different time points. Reprinted with permission.^[^ ^25g^ ^]^ Copyright 2022, American Chemical Society. (G) *T*_1_‐weighted MRI of CM@Mn with different Mn concentrations in the solution with or without GSH and (H) the corresponding *r*_1_ values. (I) In vivo *T*_1_‐weighted MRI before and after intravenous injection of @Mn or CM@Mn at different time points. Reprinted with permission.^[^ ^28^ ^]^ Copyright 2022, American Chemical Society. (J) *T*_1_‐weighted MRI of MnCl_2_ and PL/APMP with different Mn concentrations in the solution with pH 5.0 and pH 7.4 and (H) the corresponding *r*_1_ values. (L) In vivo *T*_1_‐weighted MRI before and after intravenous injection of PL/APMP at different time points. Reprinted with permission.^[^ ^107a^ ^]^ Copyright 2020, American Chemical Society.

MnO_2_, the most common form of manganese oxides used in nanomedicines, is stable under physiological conditions, but rapidly disintegrate in acidic and redox environment, thus is broadly used in TME‐responsive cancer theranostics.^[^ ^108^ ^]^ As an example, Lu's group applied MnO_2_ as carrier to load both CRISPR‐Cas9/sg‐PD‐L1 plasmid and MSA‐2 for 4T1 tumor immunotherapy. They demonstrated the prominent *T*_1_‐weighted MRI performance of the nanocomplex (HMnMPH), with *r*_1_ value counted to be 8.665 mm/s in the presence of pH 5.5 solution and 10 mm GSH. The tumor site was illuminated within 4 fours (Figure 14A–C).^[^ ^107c^ ^]^ MnO, which is less stable than MnO_2_, is more sensitive to acidity and degrades into Mn^2+^ faster in the acidic TME than MnO_2_, thus MnO is expected to be a better candidate to achieve absolute degradation in the TME and deliver abundant Mn^2+^ for tumor theranostics.^[^ ^109^ ^]^ For example, Sun and co‐workers coated MnO NPs with a hollow mesoporous silica coating and conjugated a tumor‐homing peptide iRGD (CRGDKGPD) on the NP surface as melanoma immunotheranostics.^[^ ^25g^ ^]^ The MnO dissociated in the slightly acidic TME and released Mn^2+^ for ROS production, cGAS‐STING pathway activation, and displayed *T*_1_‐weighted MRI performance, with *r*_1_ counted to be 2.48 mm/s in pH 5.4 solution (Figure 14D–F). MnO_x_ nano spikes (NSs) with mixed valence states that loaded ovalbumin and tumor cell fragments were synthesized by Ding and co‐workers.^[^ ^25c^ ^]^ The specific physical characteristic of MnO_x_ NPs rendered them as PAI agents first. The degradation of MnO_x_ in the TME promoted GSH depletion, which promoted ferroptosis. The released Mn^2+^ not only produced ROS for CDT but also enabled *T*_1_‐weighted MRI which could dynamically monitor the therapeutic process. Likewise, in a recent study, Zhao's group synthesized a MnO_x_ nanoenzyme with hybrid valences of both Mn^2+^ and Mn^3+^.^[^ ^28^ ^]^ Due to the overexpression of GSH and H_2_O_2_, all Mn^3+^ ions were transformed into Mn^2+^ ions, which manifested obvious *T*_1_‐weighted MRI performance for dynamic monitoring of the therapeutic process, with *r*_1_ calculated to be 5.78 mm/s in the presence of GSH (Figure 14G–I). In addition to manganese oxides, manganese phosphates have also been constructed to store and release Mn^2+^ in response to the TME's overexpressed GSH for *T*_1_‐weighted MRI.^[^ ^107a^ ^]^ In the study of Hou and co‐workers, the synthesized APMP NPs not only induced DNA damage, and cGAS/STING pathway activation, but also exhibited good *T*_1_‐weighted MRI performance with *r*_1_ calculated to be 4.78 mm/s in pH 5.0 solution, indicating its acidity‐responsive imaging property (Figure 14J–L).

In addition to inorganic compounds, free Mn^2+^ also coordinates with organic ligands to self‐assemble into nanoscale metal‐phenolic network The CpG‐ODN‐coated Mn‐phenolic networks (CMP) that were self‐assembled by Mn^2+^, CpG‐ODN, and TA, induced TAMs polarization and cGAS‐STING activation and further synergized with IRE for effective cancer immunotherapy.^[^ ^32^ ^]^ In this study, the research team demonstrated the effective *T*_1_‐weighted MRI performance of the CMP NPs with *r*_1_ of 11.64 mm/s, indicating the potential of the CMP NPs for image‐guided therapy. Similarly, TMA NPs that were self‐assembled by Mn^2+^, c‐di‐AMP, and TA, were validated to possess immunotherapeutic effects and *T*_1_‐weighted MRI performance.^[^ ^33^ ^]^ Mn ions have also been loaded into the phospholipid bilayer shell, with immunotheranostic potential successfully demonstrated in 4T1 tumor model.^[^ ^40^ ^]^

### 3.3 Hybrid metal‐based MSNs as immunotheranostics

**FIGURE 15:**
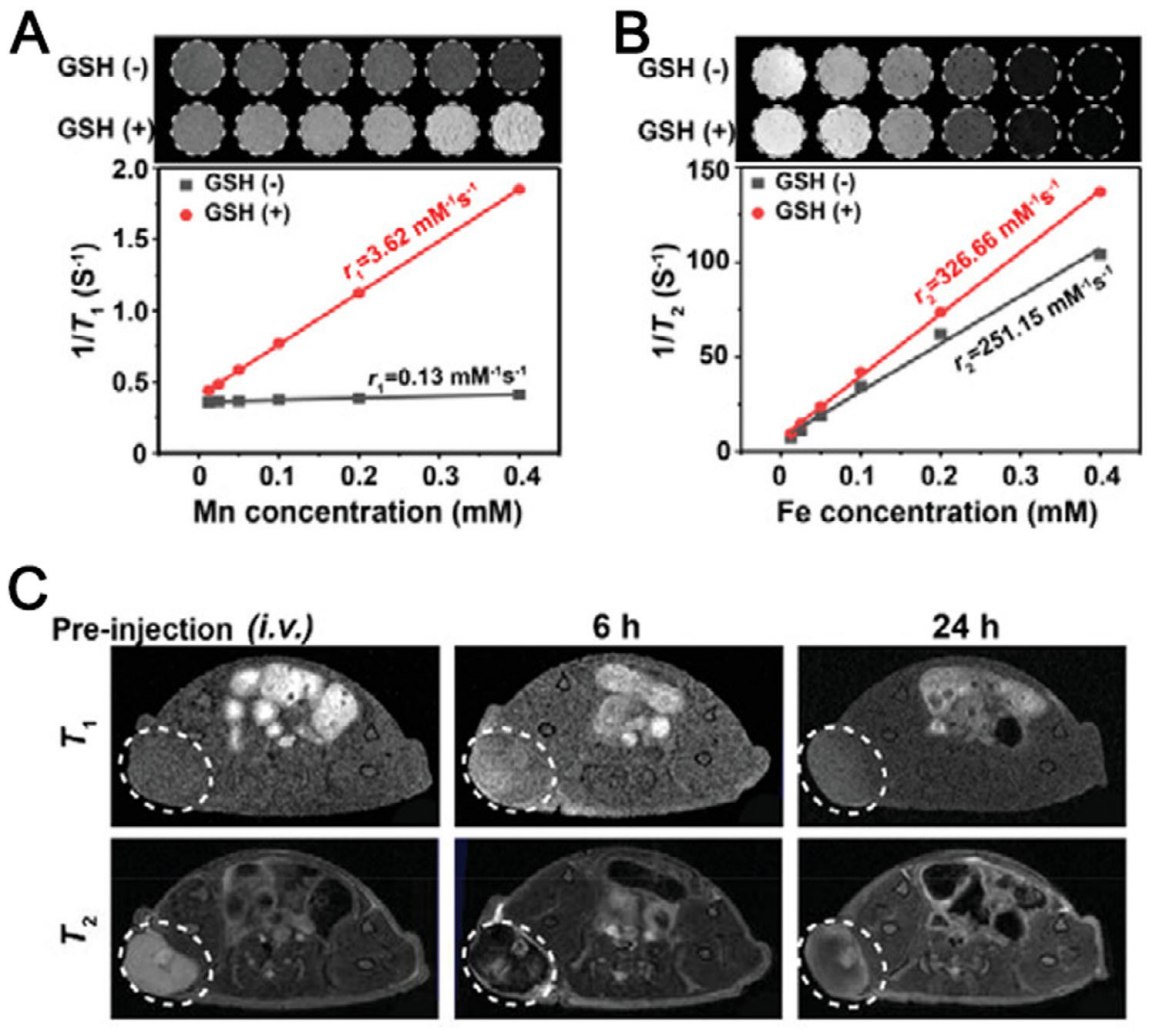
(A) *T*_1_ and (B) *T*_2_‐weighted MR images of FCMP at different Mn or Fe concentrations with or without GSH and the corresponding *r*_1_ and *r*_2_ values. (C) In vivo *T*_1_ and *T*_2_ contrast signals after intravenous injection of FCMP at different time points. Reprinted with permission. ^[^ ^24d^ ^]^ Copyright 2022, Wiley.

Since different metals possess different imaging modalities, it is interesting to explore the imaging effect induced by hybrid MSNs. To exploit the *T*_2_ weighted MRI performance of Fe_3_O_4_, and *T*_1_‐weighted MRI performance of Mn^2+^, Zhao and co‐workers constructed yolk–shell nanohybrids (Fe_3_O_4_@C/MnO_2_‐PGEA, PCMP) that were composed of Fe_3_O_4_ core, carbon interlayer, MnO_2_ shell, and a polycationic cloak.^[^ ^24d^ ^]^ To induce effective tumor inhibition, the FCMP NPs catalyzed Fenton and Fenton‐like reactions to initiate multi‐augmented CDT, and reversed the highly immunosuppressive TME by Fe‐induced TAM polarization and Mn‐induced cGAS‐STING activation. For dynamic monitoring of tumor growth, the carbon interlayer enables excellent *T*_1_ *‐T*_2_ dual‐mode MRI contrast performance to reduce the undesirable disturbance of *T*_2_ contrast agent for *T*_1_ signal, providing precise image‐guided therapy for treating various cancers (Figure 15).

## 4 CONCLUSIONS AND PERSPECTIVES

Facing the growing demand for new and effective immunotherapeutic strategies in immuno‐oncology, metalloimmunotherapy has generated great opportunities in treating highly immunosuppressive tumors, since various metal elements as we listed above (Mn, Fe, Cu, Ca, Zn, Na, Ga, Pt, K, Co, W, Rh, As, and Mo) were found to jump‐start and/or propagate the cancer immunity cycle, resulting in anti‐tumor immunity stimulation.

Regarding common chemical mechanisms of metal ion‐induced immune activity, the catalytic performance of metal ions plays prominent roles in the immune process. In our summary, the cycle between different chemical valence states of metals (Mn^2+/3+/4+^, Fe^2+/3+^, Cu^+/2+^, W^5+/6+^, Co^+/2+^, Mo^4+/6+^, As^2+/3+^) catalyzes ROS generation through Fenton or Fenton‐like reactions, thereby leading to ROS‐induced signaling pathway activation, ICD, cGAS‐STING activation, and TAM repolarization. In addition, the overload of Ca^2+^, Zn^2+^, Pt^2+^, and Na^+^ directly disrupts biological activities of cancer cells, inducing mitochondria dysfunction, endoplasmic reticulum stress, osmotic disruption, ultimately leading to ICD and immune activation. Beyond that, some metal ions exert their immunostimulatory activities by increasing sensitivity of specific enzymes, such as Mn^2+^, Zn^2+^, and Ga^3+^, thus enhancing corresponding signaling pathways and generation of downstream immunostimulatory products. Furthermore, K^+^ as summarized in our review, can even disrupt cell metabolism, thereby restricting T‐cell nutrient intake and maintain T‐cell stemness. In summary, the catalytic property, disruption of normal cell biological activities, interference of cell metabolism, and binding with specific cellular enzymes contribute to metal ion‐induced antitumor immunity.

To maximize the efficacy of metalloimmunotherapy, researchers are racing to develop highly active, specific, and flexible metal‐based nanomedicines to improve therapeutic outcomes, since inorganic nanomaterials and organic‐inorganic hybrid nanomaterials can be ideal metal nanoreservoirs to initiate metalloimmunotherapy in a TME‐responsive manner. In the TME, metal elements can promote strong antitumor immune responses via multiple pathways, including ICD induction, TAM polarization, and cGAS/STING pathway activation, thereby inhibiting tumor growth, and preventing tumor relapse and metastasis. Thanks to the flexible structure of MSNs, various components (protective coating materials, tumor‐targeting moieties, and other drugs) can be encapsulated into or conjugated onto the MSNs to achieve prolonged circulation time, enhanced tumor accumulation, and combinational therapy.

Besides, MSNs possess physical responsiveness to laser irradiation, magnetic field, ultrasound waves, and X‐rays. Therefore, physiochemical therapies, including PTT, PDT, SDT, and radiotherapy, can be applied to enhance the treatment outcome of MSNs‐led metalloimmunotherapy. Furthermore, some immunogenic MSNs with imaging functions for diagnostics can be multifunctional immunotheranostics. As a result, some critical problems in metalloimmunotherapy, such as the rapid degradation of free metals, could be largely addressed.

We preconceive that there is still a long way ahead to fully dig out the potential properties and clinical translation applications of MINs. Below we share our opinions on the critical challenges and future trends in this field.

- The antitumor immunogenic mechanisms of some metals (Mn, Fe, Cu, Ca, Zn, Na, Gd, Pt, K, W, and Co) are not fully explored at molecular level and remain controversial. For example, Fe‐based MSNs were widely known to induce antitumor immune responses through the TAM polarization pathway. However, some reported that Fe‐based MSNs polarized TAM into the M1 phenotype through the ROS‐NF‐κB pathway, while others demonstrated that it was through IRF5‐IL23 signaling pathways. Controversy also exists in Mn‐induced cGAS‐STING activation regarding whether Mn ions augment cGAMP‐STING binding affinity. The mechanism of copper‐induced antitumor effects also differs from one study to another. Copper ions have been long thought to inhibit tumor growth by CDT, thus leading to antitumor immune response, but a recent report claimed that copper led to tumor cell death through cuproptosis that was mediated by lipoylated protein aggregation and iron‐sulfur cluster protein loss.
- The immunotherapeutic potential of MSN‐induced metalloimmunotherapy was not explored thoroughly in current studies since some metal ions activate multiple immunogenic pathways to fight against tumor growth. For example, Mn ions (Mn^2+^/Mn^4+^) possess CAT‐like activity for O_2_ production and POD‐like activity for ROS production and can also affect the cGAS/STING pathway, thereby leading to TAM polarization, ICD, and type I IFN responses. However, as we summarized in this review, only one or two aspects of their immunotherapeutic potential were validated in most research. Therefore, more efforts should be devoted to exploring the therapeutic potential of metal ions to the full extent. Moreover, which mechanism plays the predominant role in the antitumor immune responses should be identified. Thus, combination therapy can be targeted to maximize overall therapeutical efficacy.
- The choice for synergistic interventions for metalloimmunotherapy is not clear. As we summarized in this review, there are a myriad of choices for combination therapies. However, a comprehensive comparison is missing among all synergistic interventions for each MSNs‐induced immunotherapy. Moreover, great heterogenicity exists among different tumor types. Therefore, for different tumor diseases, synergistic interventions should be precisely customized and individualized.
- The dynamic monitoring of the physiopathological change and therapeutic process is lacking. In terms of the immune theranostic potential of current MSNs, most studies just demonstrated the proof‐of‐principle about the imaging performance of these MSNs, such as Fe_3_O_4_ for *T*_2_‐weighted MRI, Fe^3+^/Mn^2+^/Cu^2+^/Ga^3+^ for *T*_1_‐weighted MRI. However, how we utilize the metal's imaging properties to precisely monitor real‐time therapeutic process simultaneously on a molecular level remains to be explored.
- In this review, we mainly summarized studies that intended to enhance antitumor immunity through increasing the amount of certain metal elements, we neglected the influence of metal chelation that decreased the content of certain metal elements on antitumor immunity, since there are quite a few research on the association between metal chelation and immuno‐oncology. One of the metals that is well‐explored in terms of metal content and immuno‐oncology is copper. Regarding introducing copper complexes for tumor inhibition, both anti‐inflammatory and immune‐stimulatory effects induced by copper were reported. However, regarding copper chelation, abundant studies supported the idea that copper chelation may cause immune suppression and hamper antitumor immune responsiveness. Another controversial metal is K. High intratumoral concentration of K^+^ suppressed the antitumor effect of TAMs as we summarized above. From this point, decreasing K^+^ concentration may contribute to enhanced antitumor immunity. However, in some studies, elevated intratumoral K^+^ concentration was contradictorily reported to boost antitumor immune responses (maintaining T‐cell stemness and activating cGAS‐STING signaling pathway). Therefore, the exquisite role of copper and in antitumor immunity entails in‐depth investigation.
- Last, the proper dose of MSNs needs to be determined for the optimum therapeutic outcome and minimal biological toxicity since excess metal intake would break the metal homeostasis of the body and cause unexpected side effects on major organs, such as bone marrow, spleen, liver, heart, lung, and kidneys. Most studies of MSNs‐induced metalloimmunotherapy were conducted by using animal models and the therapeutic dosage of MSNs was not well elaborated. Thus, further studies need to be done to ensure the biosafety of MSNs.

## CONFLICT OF INTEREST STATEMENT

The authors declare no conflicts of interest.

## References

[1] R. S. Riley , C. H. June , R. Langer , M. J. Mitchell , Nat. Rev. Drug Discovery 2019, 18, 175. 30622344 10.1038/s41573-018-0006-z PMC6410566

[2] Y. Zhang , Z. Zhang , Cell Mol. Immunol. 2020, 17, 807. 32612154 10.1038/s41423-020-0488-6 PMC7395159

[3] D. R. Wang , X. L. Wu , Y. L. Sun , Signal Transduct. Target Ther. 2022, 7, 331. 36123348 10.1038/s41392-022-01136-2 PMC9485144

[4] P. S. Hegde , D. S. Chen , Immunity 2020, 52, 17. 31940268 10.1016/j.immuni.2019.12.011

[5] E. J. M. Huijbers , K. A. Khan , R. S. Kerbel , A. W. Griffioen , Sci. Immunol. 2022, 7, eabm6388. 35030032 10.1126/sciimmunol.abm6388

[6] S. Bagchi , R. Yuan , E. G. Engleman , Annu. Rev. Pathol. 2021, 16, 223. 33197221 10.1146/annurev-pathol-042020-042741

[7] M. de Miguel , E. Calvo ,Cancer Cell 2020, 38, 326. 32750319 10.1016/j.ccell.2020.07.004

[8] B. Englinger , C. Pirker , P. Heffeter , A. Terenzi , C. R. Kowol , B. K. Keppler , W. Berger , Chem. Rev. 2019, 119, 1519. 30489072 10.1021/acs.chemrev.8b00396

[9] C. Wang , R. Zhang , X. Wei , M. Lv , Z. Jiang , Adv. Immunol. 2020, 145, 187. 32081198 10.1016/bs.ai.2019.11.007

[10] a) W. Cao , M. Jin , K. Yang , B. Chen , M. Xiong , X. Li , G. Cao , J. Nanobiotechnol. 2021, 19, 325; 10.1186/s12951-021-01074-1 PMC8520258 34656118

[11] a) M. Lv , M. Chen , R. Zhang , W. Zhang , C. Wang , Y. Zhang , X. Wei , Y. Guan , J. Liu , K. Feng , M. Jing , X. Wang , Y. C. Liu , Q. Mei , W. Han , Z. Jiang , Cell Res. 2020, 30, 966; 32839553 10.1038/s41422-020-00395-4 PMC7785004

[12] a) J. An , K. Zhang , B. Wang , S. Wu , Y. Wang , H. Zhang , Z. Zhang , J. Liu , J. Shi , ACS Nano 2020, 14, 7639; 32427462 10.1021/acsnano.0c03881

[13] W. Jiang , L. Yin , H. Chen , A. V. Paschall , L. Zhang , W. Fu , W. Zhang , T. Todd , K. S. Yu , S. Zhou , Z. Zhen , M. Butler , L. Yao , F. Zhang , Y. Shen , Z. Li , A. Yin , H. Yin , X. Wang , F. Y. Avci , X. Yu , J. Xie , Adv. Mater. 2019, 31, e1904058. 31553099 10.1002/adma.201904058 PMC6886716

[14] J. J. Kim , Y. S. Kim , V. Kumar , J. Trace Elem. Med. Biol. 2019, 54, 226. 31109617 10.1016/j.jtemb.2019.05.003

[15] Q. Peña , A. Wang , O. Zaremba , Y. Shi , H. W. Scheeren , J. M. Metselaar , F. Kiessling , R. M. Pallares , S. Wuttke , T. Lammers , Chem. Soc. Rev. 2022, 51, 2544. 35262108 10.1039/d1cs00468a

[16] S. Sen , M. Won , M. S. Levine , Y. Noh , A. C. Sedgwick , J. S. Kim , J. L. Sessler , J. F. Arambula , Chem. Soc. Rev. 2022, 51, 1212. 35099487 10.1039/d1cs00417d PMC9398513

[17] R. Singh , A. Sharma , J. Saji , A. Umapathi , S. Kumar , H. K. Daima , Nano Converg. 2022, 9, 21. 35569081 10.1186/s40580-022-00313-x PMC9108129

[18] a) A. Mohapatra , P. Sathiyamoorthy , I. K. Park , Pharmaceutics 2021, 13, 1867; 34834282 10.3390/pharmaceutics13111867 PMC8622235

[19] R. M. Hooy , G. Massaccesi , K. E. Rousseau , M. A. Chattergoon , J. Sohn , Nucleic Acids Res. 2020, 48, 4435. 32170294 10.1093/nar/gkaa084 PMC7192592

[20] K. Zhang , C. Qi , K. Cai , Adv. Mater. 2022, 35, e2205409. 10.1002/adma.202205409 36121368

[21] X. Sun , Y. Zhang , J. Li , K. S. Park , K. Han , X. Zhou , Y. Xu , J. Nam , J. Xu , X. Shi , L. Wei , Y. L. Lei , J. J. Moon , Nat. Nanotechnol. 2021, 16, 1260. 34594005 10.1038/s41565-021-00962-9 PMC8595610

[22] K. M. Garland , T. L. Sheehy , J. T. Wilson , Chem. Rev. 2022, 122, 5977. 35107989 10.1021/acs.chemrev.1c00750 PMC8994686

[23] C. Zhao , H. Deng , X. Chen , Adv. Drug Delivery Rev. 2022, 188, 114456. 10.1016/j.addr.2022.114456 35843505

[24] a) M. Song , T. Liu , C. Shi , X. Zhang , X. Chen , ACS Nano 2016, 10, 633; 26650065 10.1021/acsnano.5b06779 PMC5242343

[25] a) G. Yang , L. Xu , Y. Chao , J. Xu , X. Sun , Y. Wu , R. Peng , Z. Liu , Nat. Commun. 2017, 8, 902; 29026068 10.1038/s41467-017-01050-0 PMC5638920

[26] Y. Zhao , Y. Pan , K. Zou , Z. Lan , G. Cheng , Q. Mai , H. Cui , Q. Meng , T. Chen , L. Rao , L. Ma , G. Yu , Bioact. Mater. 2023, 19, 237. 35510176 10.1016/j.bioactmat.2022.04.011 PMC9048124

[27] G. Hou , J. Qian , M. Guo , W. Xu , J. Wang , Y. Wang , A. Suo , J. Colloid Interface Sci. 2022, 628, 968. 36037718 10.1016/j.jcis.2022.08.091

[28] Z. Zhao , S. Dong , Y. Liu , J. Wang , L. Ba , C. Zhang , X. Cao , C. Wu , P. Yang , ACS Nano 2022, 16, 20400. 36441901 10.1021/acsnano.2c06646

[29] Z. Li , Z. Chu , J. Yang , H. Qian , J. Xu , B. Chen , T. Tian , H. Chen , Y. Xu , F. Wang , ACS Nano 2022, 16, 15471. 35981098 10.1021/acsnano.2c08013

[30] L. Huang , Y. Liao , C. Li , Z. Ma , Z. Liu , Biomater. Adv. 2022, 136, 212752. 35929287 10.1016/j.bioadv.2022.212752

[31] a) Y. Guo , Q. Sun , F. G. Wu , Y. Dai , X. Chen , Adv. Mater. 2021, 33, e2007356; 33876449 10.1002/adma.202007356

[32] J. H. Han , H. E. Shin , J. Lee , J. M. Kang , J. H. Park , C. G. Park , D. K. Han , I. H. Kim , W. Park , Small 2022, 18, e2200316. 35570584 10.1002/smll.202200316

[33] D. Wang , T. Nie , C. Huang , Z. Chen , X. Ma , W. Fang , Y. Huang , L. Luo , Z. Xiao , Small 2022, 18, e2203227. 36026551 10.1002/smll.202203227

[34] J. Li , H. Ren , Q. Qiu , X. Yang , J. Zhang , C. Zhang , B. Sun , J. F. Lovell , Y. Zhang , ACS Nano 2022, 16, 16909. 36200692 10.1021/acsnano.2c06926

[35] C. Chen , Y. Tong , Y. Zheng , Y. Shi , Z. Chen , J. Li , X. Liu , D. Zhang , H. Yang , Small 2021, 17, e2006970. 33719177 10.1002/smll.202006970

[36] X. Wang , Y. Liu , C. Xue , Y. Hu , Y. Zhao , K. Cai , M. Li , Z. Luo , Nat. Commun. 2022, 13, 5685. 36167857 10.1038/s41467-022-33301-0 PMC9515186

[37] X. Zhao , Y. Wang , W. Jiang , Q. Wang , J. Li , Z. Wen , A. Li , K. Zhang , Z. Zhang , J. Shi , J. Liu , Adv. Mater. 2022, 34, e2204585. 35869026 10.1002/adma.202204585

[38] S. J. Zheng , M. Yang , J. Q. Luo , R. Liu , J. Song , Y. Chen , J. Z. Du , ACS Nano 2023, 17, 15905. 37565626 10.1021/acsnano.3c03962

[39] Z. L. Gao , W. Xu , S. J. Zheng , Q. J. Duan , R. Liu , J. Z. Du , Nano Lett. 2023, 23, 1904. 36801829 10.1021/acs.nanolett.2c04970

[40] C. Zhu , Q. Ma , L. Gong , S. Di , J. Gong , Y. Wang , S. Xiao , L. Zhang , Q. Zhang , J. J. Fu , D. Lu , Z. Lin , Acta Biomater. 2022, 141, 429. 35038584 10.1016/j.actbio.2022.01.019

[41] a) Q. Jiang , K. Wang , X. Zhang , B. Ouyang , H. Liu , Z. Pang , W. Yang , Small 2020, 16, e2001704; 32338436 10.1002/smll.202001704

[42] a) P. Guo , L. Wang , W. Shang , J. Chen , Z. Chen , F. Xiong , Z. Wang , Z. Tong , K. Wang , L. Yang , J. Tian , W. Xu , ACS Appl. Mater. Interfaces 2020, 12, 54367; 33236624 10.1021/acsami.0c15176

[43] M. Weiss , K. Blazek , A. J. Byrne , D. P. Perocheau , I. A. Udalova , Mediators Inflamm. 2013, 2013, 245804. 24453413 10.1155/2013/245804 PMC3885211

[44] a) L. Liu , Y. Wang , X. Guo , J. Zhao , S. Zhou , Small 2020, 16, e2003543; 32812355 10.1002/smll.202003543

[45] S. Chung , R. A. Revia , M. Zhang , Nanoscale Horiz. 2021, 6, 696. 34286791 10.1039/d1nh00179e PMC8496976

[46] a) G.‐T. Yu , L. Rao , H. Wu , L.‐L. Yang , L.‐L. Bu , W.‐W. Deng , L. Wu , X. Nan , W.‐F. Zhang , X.‐Z. Zhao , W. Liu , Z.‐J. Sun , Adv. Funct. Mater. 2018, 28, 1801389;

[47] S. Fu , R. Yang , J. Ren , J. Liu , L. Zhang , Z. Xu , Y. Kang , P. Xue , ACS Nano 2021, 15, 11953. 34142808 10.1021/acsnano.1c03128

[48] Q. Xiang , C. Yang , Y. Luo , F. Liu , J. Zheng , W. Liu , H. Ran , Y. Sun , J. Ren , Z. Wang , Small 2022, 18, e2107809. 35143709 10.1002/smll.202107809

[49] X. Wang , Y. Cheng , X. Han , J. Yan , Y. Wu , P. Song , Y. Wang , X. Li , H. Zhang , Adv. Healthc. Mater. 2022, 11, e2200776. 35912918 10.1002/adhm.202200776

[50] M. Ding , W. Liu , R. Gref , Adv. Drug Delivery Rev. 2022, 190, 114496. 10.1016/j.addr.2022.114496 35970275

[51] Y. Yu , Z. Huang , Q. Chen , Z. Zhang , H. Jiang , R. Gu , Y. Ding , Y. Hu , Biomaterials 2022, 288, 121724. 36038420 10.1016/j.biomaterials.2022.121724

[52] Y. Gao , G. Yu , K. Xing , D. Gorin , Y. Kotelevtsev , W. Tong , Z. Mao , J. Mater. Chem. B. 2020, 8, 7121. 32648878 10.1039/d0tb01248c

[53] K. Sun , J. Yu , J. Hu , J. Chen , J. Song , Z. Chen , Z. Cai , Z. Lu , L. Zhang , Z. Wang , Acta Biomater. 2022, 148, 230. 35724919 10.1016/j.actbio.2022.06.026

[54] Z. Wang , Y. Zou , Y. Li , Y. Cheng , Small 2020, 16, e1907042. 32220006 10.1002/smll.201907042

[55] L. Rong , Y. Zhang , W. S. Li , Z. Su , J. I. Fadhil , C. Zhang , Biomaterials 2019, 225, 119515. 31590119 10.1016/j.biomaterials.2019.119515

[56] N. Singh , J. Kim , J. Kim , K. Lee , Z. Zunbul , I. Lee , E. Kim , S. G. Chi , J. S. Kim , Bioact. Mater. 2023, 21, 358. 36185736 10.1016/j.bioactmat.2022.08.016 PMC9483748

[57] D. Wang , L. Lin , T. Li , M. Meng , K. Hao , Z. Guo , J. Chen , H. Tian , X. Chen , Adv. Mater. 2022, 34, e2205924. 36039617 10.1002/adma.202205924

[58] A. Pal , F. Arshad , M. P. Sk , Adv. Colloid Interface Sci. 2020, 285, 102274. 32992078 10.1016/j.cis.2020.102274

[59] Y. Sang , Q. Deng , F. Cao , Z. Liu , Y. You , H. Liu , J. Ren , X. Qu , ACS Nano 2021, 15, 19298. 34783526 10.1021/acsnano.1c05392

[60] Y. Liu , Y. Wang , C. Wang , T. Dong , H. Xu , Y. Guo , X. Zhao , Y. Hu , J. Wu , Adv. Sci. 2022, 9, e2203027. 10.1002/advs.202203027 PMC9631083 36073796

[61] L. Hou , D. Chen , R. Wang , R. Wang , H. Zhang , Z. Zhang , Z. Nie , S. Lu , Angew. Chem., Int. Ed. 2021, 60, 6581. 10.1002/anie.202014397 33305859

[62] F. Duan , W. Jin , T. Zhang , F. Zhang , L. Gong , X. Liu , X. Deng , W. Gao , ACS Appl. Mater. Interfaces 2022, 14, 32823. 10.1021/acsami.2c04894 35849733

[63] B. Xu , Y. Cui , W. Wang , S. Li , C. Lyu , S. Wang , W. Bao , H. Wang , M. Qin , Z. Liu , W. Wei , H. Liu , Adv. Mater. 2020, 32, e2003563. 32627937 10.1002/adma.202003563

[64] Y. Wang , Y. Chen , J. Zhang , Y. Yang , J. S. Fleishman , Y. Wang , J. Wang , J. Chen , Y. Li , H. Wang , Drug Resist. Updat. 2023, 72, 101018. 37979442 10.1016/j.drup.2023.101018

[65] P. Tsvetkov , S. Coy , B. Petrova , M. Dreishpoon , A. Verma , M. Abdusamad , J. Rossen , L. Joesch‐Cohen , R. Humeidi , R. D. Spangler , J. K. Eaton , E. Frenkel , M. Kocak , S. M. Corsello , S. Lutsenko , N. Kanarek , S. Santagata , T. R. Golub , Science 2022, 375, 1254. 35298263 10.1126/science.abf0529 PMC9273333

[66] X. Yu , Y. Wang , J. Zhang , J. Liu , A. Wang , L. Ding , Adv. Healthc. Mater. 2023, 13, e2302023. 37742127 10.1002/adhm.202302023

[67] W. Zeng , M. Yu , T. Chen , Y. Liu , Y. Yi , C. Huang , J. Tang , H. Li , M. Ou , T. Wang , M. Wu , L. Mei , Adv. Sci. 2022, 9, e2201703. 10.1002/advs.202201703 PMC9376744 35678111

[68] C. Xu , Y. Liu , Y. Zhang , L. Gao , Front. Genet. 2022, 13, 928105. 36313449 10.3389/fgene.2022.928105 PMC9596916

[69] Y. Zheng , Y. Han , T. Wang , H. Liu , Q. Sun , S. Hu , J. Chen , Z. Li , Adv. Funct. Mater. 2022, 32, 2108971.

[70] L. Qiao , G. Zhu , T. Jiang , Y. Qian , Q. Sun , G. Zhao , H. Gao , C. Li , Adv. Mater. 2023, 36, e2308241. 37820717 10.1002/adma.202308241

[71] X.‐K. Jin , J.‐L. Liang , S.‐M. Zhang , Q.‐X. Huang , S.‐K. Zhang , C.‐J. Liu , X.‐Z. Zhang , Mater. Today 2023, 68, 108.

[72] M. Chang , M. Wang , M. Wang , M. Shu , B. Ding , C. Li , M. Pang , S. Cui , Z. Hou , J. Lin , Adv. Mater. 2019, 31, e1905271. 31680346 10.1002/adma.201905271

[73] X. Zhang , S. Wang , K. Tang , W. Pan , H. Xu , Y. Li , Y. Gao , N. Li , B. Tang , ACS Appl. Mater. Interfaces 2022, 14, 30618. 35763788 10.1021/acsami.2c07739

[74] L. Zhang , Q. C. Yang , S. Wang , Y. Xiao , S. C. Wan , H. Deng , Z. J. Sun , Adv. Mater. 2022, 34, e2108174. 34918837 10.1002/adma.202108174

[75] M. Wang , M. Chang , C. Li , Q. Chen , Z. Hou , B. Xing , J. Lin , Adv. Mater. 2022, 34, e2106010. 34699627 10.1002/adma.202106010

[76] S. Chattopadhyay , Y. H. Liu , Z. S. Fang , C. L. Lin , J. C. Lin , B. Y. Yao , C. J. Hu , Nano Lett. 2020, 20, 2246. 32160474 10.1021/acs.nanolett.9b04094

[77] a) E. Bertero , C. Maack , Circ. Res. 2018, 122, 1460; 29748369 10.1161/CIRCRESAHA.118.310082

[78] a) D. L. Medina , A. Ballabio , Autophagy 2015, 11, 970; 26000950 10.1080/15548627.2015.1047130 PMC4502748

[79] C. Zheng , Q. Song , H. Zhao , Y. Kong , L. Sun , X. Liu , Q. Feng , L. Wang , J. Control Release. 2021, 339, 403. 34655676 10.1016/j.jconrel.2021.10.011

[80] a) C. Hübner , H. Haase , Redox Biol. 2021, 41, 101916; 33662875 10.1016/j.redox.2021.101916 PMC7937829

[81] Y. Zhang , C. Guo , L. Liu , J. Xu , H. Jiang , D. Li , J. Lan , J. Li , J. Yang , Q. Tu , X. Sun , M. Alamgir , X. Chen , G. Shen , J. Zhu , J. Tao , Theranostics 2020, 10, 11197. 33042278 10.7150/thno.44920 PMC7532661

[82] Z. Dai , Q. Wang , J. Tang , M. Wu , H. Li , Y. Yang , X. Zhen , C. Yu , Biomaterials 2022, 280, 121261. 34815099 10.1016/j.biomaterials.2021.121261

[83] L. Zhang , J. Zhao , X. Hu , C. Wang , Y. Jia , C. Zhu , S. Xie , J. Lee , F. Li , D. Ling , Adv. Mater. 2022, 34, e2206915. 35986645 10.1002/adma.202206915

[84] D. Cen , Q. Ge , C. Xie , Q. Zheng , J. Guo , Y. Zhang , Y. Wang , X. Li , Z. Gu , X. Cai , Adv. Mater. 2021, 33, e2104037. 34622500 10.1002/adma.202104037

[85] S. F. Pedersen , A. Kapus , E. K. Hoffmann , J. Am. Soc. Nephrol. 2011, 22, 1587. 21852585 10.1681/ASN.2010121284

[86] a) Y. Liu , W. Zhen , Y. Wang , S. Song , H. Zhang , J. Am. Chem. Soc. 2020, 142, 21751; 33337859 10.1021/jacs.0c09482

[87] X. Wei , F. Xie , X. Zhou , Y. Wu , H. Yan , T. Liu , J. Huang , F. Wang , F. Zhou , L. Zhang , Cell. Mol. Immunol. 2022, 19, 971. 35970871 10.1038/s41423-022-00905-x PMC9376585

[88] R. B. Birge , S. Boeltz , S. Kumar , J. Carlson , J. Wanderley , D. Calianese , M. Barcinski , R. A. Brekken , X. Huang , J. T. Hutchins , B. Freimark , C. Empig , J. Mercer , A. J. Schroit , G. Schett , M. Herrmann , Cell Death Differ. 2016, 23, 962. 26915293 10.1038/cdd.2016.11 PMC4987730

[89] M. Rogosnitzky , S. Branch , Biometals 2016, 29, 365. 27053146 10.1007/s10534-016-9931-7 PMC4879157

[90] L. Cao , H. Tian , M. Fang , Z. Xu , D. Tang , J. Chen , J. Yin , H. Xiao , K. Shang , H. Han , X. Li , Biomaterials 2022, 290, 121856. 36306685 10.1016/j.biomaterials.2022.121856

[91] P. W. Shueng , L. Y. Yu , H. C. Chiu , H. C. Chang , Y. L. Chiu , T. Y. Kuo , Y. W. Yen , C. L. Lo , Biomaterials 2021, 276, 121012. 34252800 10.1016/j.biomaterials.2021.121012

[92] I. Banerjee , B. Behl , M. Mendonca , G. Shrivastava , A. J. Russo , A. Menoret , A. Ghosh , A. T. Vella , S. K. Vanaja , S. N. Sarkar , K. A. Fitzgerald , V. A. K. Rathinam , Immunity 2018, 49, 413. 30170814 10.1016/j.immuni.2018.07.006 PMC6347470

[93] a) S. K. Vodnala , R. Eil , R. J. Kishton , M. Sukumar , T. N. Yamamoto , N. H. Ha , P. H. Lee , M. Shin , S. J. Patel , Z. Yu , D. C. Palmer , M. J. Kruhlak , X. Liu , J. W. Locasale , J. Huang , R. Roychoudhuri , T. Finkel , C. A. Klebanoff , N. P. Restifo , Science 2019, 363, eaau0135; 30923193 10.1126/science.aau0135 PMC8194369

[94] S. Chen , W. Cui , Z. Chi , Q. Xiao , T. Hu , Q. Ye , K. Zhu , W. Yu , Z. Wang , C. Yu , X. Pan , S. Dai , Q. Yang , J. Jin , J. Zhang , M. Li , D. Yang , Q. Yu , Q. Wang , X. Yu , W. Yang , X. Zhang , J. Qian , K. Ding , D. Wang , Cell Metab. 2022, 34, 1843. 36103895 10.1016/j.cmet.2022.08.016

[95] a) N. Toupin , M. K. Herroon , R. P. Thummel , C. Turro , I. Podgorski , H. Gibson , J. J. Kodanko , Chemistry 2022, 28, e202104430; 35235227 10.1002/chem.202104430 PMC9541094

[96] a) V. Bordignon , F. Palamara , P. Cordiali‐Fei , A. Vento , A. Aiello , M. Picardo , F. Ensoli , A. Cristaudo , BMC Immunol. 2008, 9, 19; 18482439 10.1186/1471-2172-9-19 PMC2409297

[97] Q. Q. Wang , Y. Jiang , H. Naranmandura , Metallomics 2020, 12, 326. 32163072 10.1039/c9mt00308h

[98] a) Y. Hu , J. Liang , Y. Xia , C. Zhao , M. Jiang , J. Ma , Z. Tie , Z. Jin , Small 2022, 18, e2104556; 34846791 10.1002/smll.202104556

[99] X. Wang , J. Zhang , Y. Hu , X. Zhao , Z. Wang , W. Zhang , J. Liang , W. Yu , T. Tian , H. Zhou , J. Li , S. Liu , J. Zhao , Z. Jin , W. Wei , Z. Guo , ACS Appl. Mater. Interfaces 2022, 14, 45137. 36166745 10.1021/acsami.2c10743

[100] N. Dhas , R. Kudarha , A. Garkal , V. Ghate , S. Sharma , P. Panzade , S. Khot , P. Chaudhari , A. Singh , M. Paryani , S. Lewis , N. Garg , N. Singh , P. Bangar , T. Mehta , J. Control Release 2021, 330, 257. 33345832 10.1016/j.jconrel.2020.12.015

[101] S. Roy , K. A. Deo , K. A. Singh , H. P. Lee , A. Jaiswal , A. K. Gaharwar , Adv. Drug Delivery Rev. 2022, 187, 114361. 10.1016/j.addr.2022.114361 PMC12861116 35636569

[102] a) R. Cui , L. Wang , D. Zhang , K. Zhang , J. Dou , L. Dong , Y. Zhang , J. Wu , L. Tan , J. Yu , P. Liang , Acta Pharm. Sin. B. 2022, 12, 3475; 36176908 10.1016/j.apsb.2022.05.026 PMC9513490

[103] a) J. Zhou , D. Guo , Y. Zhang , W. Wu , H. Ran , Z. Wang , ACS Appl. Mater. Interfaces 2014, 6, 5566; 24693875 10.1021/am406008k

[104] S. Y. Srinivasan , K. M. Paknikar , D. Bodas , V. Gajbhiye , Nanomedicine 2018, 13, 1221. 29882719 10.2217/nnm-2017-0379

[105] P. Zhang , Y. Hou , J. Zeng , Y. Li , Z. Wang , R. Zhu , T. Ma , M. Gao , Angew. Chem., Int. Ed. 2019, 58, 11088. 10.1002/anie.201904880 31131511

[106] a) R. Zheng , J. Guo , X. Cai , L. Bin , C. Lu , A. Singh , M. Trivedi , A. Kumar , J. Liu , Colloids Surf. B Biointerfaces 2022, 213, 112432; 35259704 10.1016/j.colsurfb.2022.112432

[107] a) L. Hou , C. Tian , Y. Yan , L. Zhang , H. Zhang , Z. Zhang , ACS Nano 2020, 14, 3927; 32298077 10.1021/acsnano.9b06111

[108] a) B. Ding , P. Zheng , P. Ma , J. Lin , Adv. Mater. 2020, 32, e1905823; 31990409 10.1002/adma.201905823

[109] a) Q. Sun , J. Yang , W. Shen , H. Lu , X. Hou , Y. Liu , Y. Xu , Q. Wu , Z. Xuan , Y. Yang , D. Yin , Biomaterials 2022, 289, 121796; 36108581 10.1016/j.biomaterials.2022.121796

